# Using Multi-Compartment Ensemble Modeling As an Investigative Tool of Spatially Distributed Biophysical Balances: Application to Hippocampal Oriens-Lacunosum/Moleculare (O-LM) Cells

**DOI:** 10.1371/journal.pone.0106567

**Published:** 2014-10-31

**Authors:** Vladislav Sekulić, J. Josh Lawrence, Frances K. Skinner

**Affiliations:** 1 Toronto Western Research Institute, University Health Network, Toronto, Ontario, Canada; 2 Department of Physiology, University of Toronto, Toronto, Ontario, Canada; 3 Department of Medicine (Neurology), University of Toronto, Toronto, Ontario, Canada; 4 NIH COBRE Center for Structural and Functional Neuroscience, University of Montana, Missoula, Montana, United States of America; 5 Department of Biomedical and Pharmaceutical Sciences, University of Montana, Missoula, Montana, United States of America; Federal University of Rio Grande do Norte, Brazil

## Abstract

Multi-compartmental models of neurons provide insight into the complex, integrative properties of dendrites. Because it is not feasible to experimentally determine the exact density and kinetics of each channel type in every neuronal compartment, an essential goal in developing models is to help characterize these properties. To address biological variability inherent in a given neuronal type, there has been a shift away from using hand-tuned models towards using ensembles or populations of models. In collectively capturing a neuron's output, ensemble modeling approaches uncover important conductance balances that control neuronal dynamics. However, conductances are never entirely known for a given neuron class in terms of its types, densities, kinetics and distributions. Thus, any multi-compartment model will always be incomplete. In this work, our main goal is to use ensemble modeling as an investigative tool of a neuron's biophysical balances, where the cycling between experiment and model is a design criterion from the start. We consider oriens-lacunosum/moleculare (O-LM) interneurons, a prominent interneuron subtype that plays an essential gating role of information flow in hippocampus. O-LM cells express the hyperpolarization-activated current (*I*_h_). Although dendritic *I*_h_ could have a major influence on the integrative properties of O-LM cells, the compartmental distribution of *I*_h_ on O-LM dendrites is not known. Using a high-performance computing cluster, we generated a database of models that included those with or without dendritic *I*_h_. A range of conductance values for nine different conductance types were used, and different morphologies explored. Models were quantified and ranked based on minimal error compared to a dataset of O-LM cell electrophysiological properties. Co-regulatory balances between conductances were revealed, two of which were dependent on the presence of dendritic *I*_h_. These findings inform future experiments that differentiate between somatic and dendritic *I*_h_, thereby continuing a cycle between model and experiment.

## Introduction

Neurons possess diverse, elaborate morphologies that allow the propagation of electrical signals through fine, intricate neuronal structures [1]. Due to the pioneering work of Rall [2], many multi-compartment mathematical models of different neurons have been developed. Why do we build these models and what are they good for? The answer to this of course depends on the modeling goals in general, and specifically, on how the model will be used. Certainly, having multi-compartment models in hand allows us to explore aspects not possible or feasible to do experimentally, and to provide mechanistic insights into experimental observations and paradoxical results [3], [4]. Nevertheless, our developed models are limited by the lack of knowledge of the biological details. That is, for any given neuron type, there are inevitable uncertainties regarding the properties of each electrical compartment of the modeled neuron that cannot be inferred from experimental observations alone. Therefore, the use of multi-compartment models allows us to simulate different possibilities in terms of ion channel types and properties in order to test plausible mechanisms of neuronal function and generate predictions that can be experimentally examined.

An essential goal when building multi-compartment models of neurons is to understand how the density, kinetics and distribution of biophysical conductances give rise to the neuron's observed dynamical output. Given the nonlinear and multi-faceted nature of neuronal dynamics, such a goal requires insight obtained from analyzing developed models. However, with the high-dimensionality of multi-compartment model equations, mathematical analysis tools are not available. Furthermore, it has become clear that several ion channel types across multiple vertebrate and invertebrate species have varying conductances for a given cell type [5]. For cells to maintain consistent output across this variability in intrinsic conductances, different conductances could balance against each other in a homeostatic way to allow this as required for network functioning [6]. This principle was conclusively demonstrated in a recent study [7]. A clear implication of this variability is that it is difficult to consider that a single, biophysically-based multi-compartment model can capture the experimental data. As such, there has been a shift towards developing populations, or ensembles of models that can collectively capture a neuron's physiological essence. Analysis of these model ensembles can subsequently reveal compensatory balances of conductances [8], and in this way, ensemble modeling can be considered as a form of analysis for multi-compartment models.

Inhibitory cells are a heterogeneous class of neurons [9], [10]. Their cell type-specific characteristics are likely to be functionally important [11]–[14]. Among the various interneuron subtypes in the hippocampus, the oriens-lacunosum/moleculare (O-LM) cell located in the CA1 region plays an important role in information flow, neuronal rhythms, and synaptic plasticity [9], [14]–[16]. O-LM cells possess a distinct morphology. Their somata and dendrites are located in the stratum oriens layer. In contrast, their dense axonal arborizations project to the lacunosum/moleculare layer, synapsing onto distal dendrites of pyramidal cells, where they are thought to influence the efficacy of perforant path input [17], [18]. O-LM cells have a variety of voltage-gated ion channels across their somatodendritic and axonal compartments. In particular, one hallmark feature of O-LM cells is that they exhibit a “sag” response to hyperpolarizing current steps, which indicates the presence of hyperpolarization-activated cation currents or *I*_h_ [19]. *I*_h_ contributes to the spontaneous firing of O-LM cells allowing them to play a pacemaker role, and models have suggested how they could contribute to the generation of population theta activities ([20], but see [21]). While the existence of *I*_h_ in O-LM cells has been known for well over a decade, it is unknown whether *I*_h_ is present in the dendrites of O-LM cells. This is essential to consider as the integrative properties of the dendritic tree in response to synaptic input could be modulated by dendritic *I*_h_, and given that synaptic plasticity is known to exist for these cell types [9], [15], this is even more critical. Unfortunately, in general, performing non-somatic recordings on specific cell types is a difficult endeavor in rats, and is particularly challenging in mice. Although multi-compartment O-LM cell models that capture salient features of experimental data have been developed [22]–[24], they only include somatically located *I*_h_. Thus, obtaining any insights from modeling work would be beneficial.

**Figure 1:**
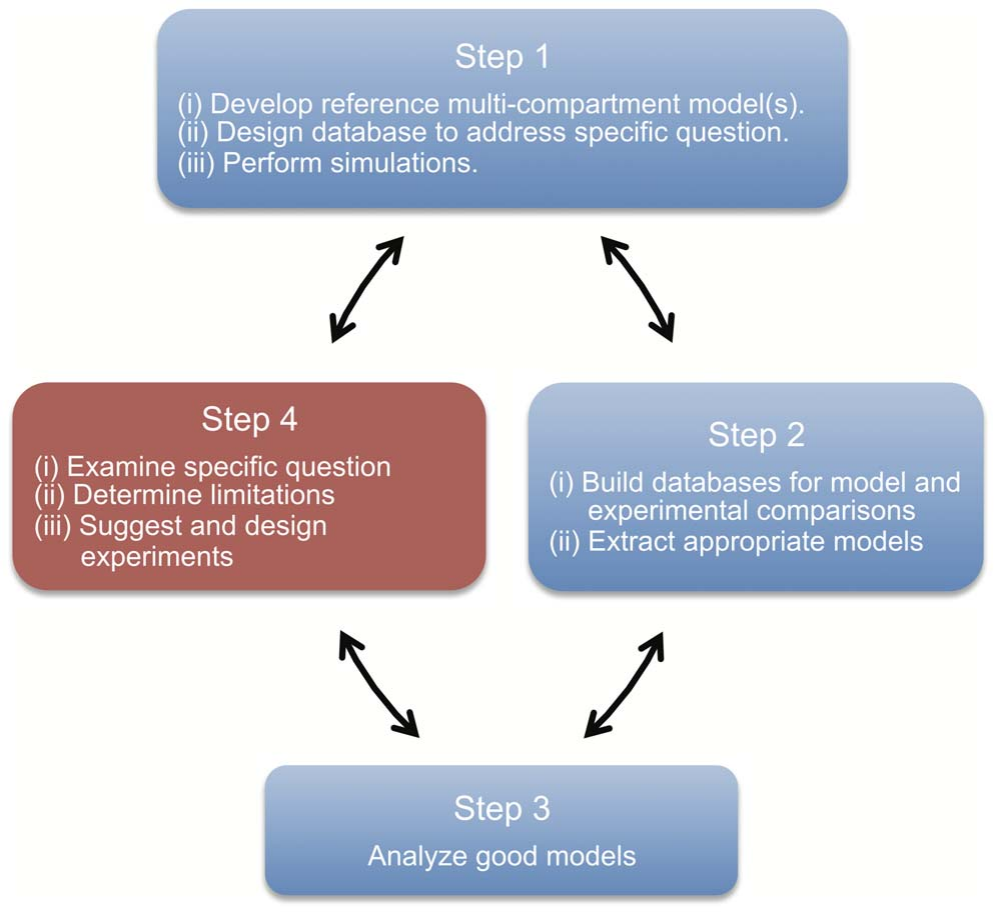
The cyclical ensemble modeling approach. Schematic highlighting the methodological links between stages in the ensemble modeling approach.

In this paper, we apply ensemble modeling to hippocampal O-LM cells in order to consider dendritic *I*_h_. In doing this, we propose an *experiment-modeling cycling* approach (Fig. 1). The benefit of ensemble modeling has been demonstrated [5]–[8]. Our intent with the cycling approach here is to take advantage of it in the context of hippocampal interneurons. Importantly, we focus on multi-compartment models to allow consideration of non-somatic properties since, experimentally, this is where the most challenging aspects lie, and where functionally relevant aspects due to cellular and synaptic network interactions matter. A major motivation in our approach is to solidify what should be the best “next step” to take in consideration of detailed, multi-compartment models. Although more detail can always be added, having a basis or rationale of what would make the most sense to consider next is part of what underlies our approach. The cycling involves: (1) model development, database design and simulations, (2) database building and model extraction, (3) model analysis, and (4) design examination, limitation determination and back to model development, as schematized in Fig. 1.

In the present paper, the database design is focused on examining whether *I*_h_ is present in the dendrites of O-LM cells. We use experimental recordings to statistically rank and extract a subset of O-LM models that conform to O-LM cell output. We analyze the resulting ensemble using techniques for the visualization of high-dimensional parameter spaces, and examining conductance histogram plots. We find three co-regulatory conductance balances, two of which are dependent on the presence of dendritic *I*_h_. Particular experiments are proposed as well as consideration of possible next steps.

## Results

### A methodology for cycling between experiment and modeling to investigate distributed biophysical conductances in multi-compartment neuronal models

Developing detailed, multi-compartment neuron models is a commitment. While it is clear that such models are needed to help us understand many aspects in the nervous system, it is far from clear how much detail one should – or needs to – include in such models. Once a detailed, multi-compartment model is in hand, the question of its biological correctness and appropriateness is an evolving process, especially concerning densities, kinetics, and distributions of voltage-gated ion channels on neuronal dendrites. It is clear that one cannot take a purely mathematical approach with such models as not only are there many model parameter values that need to be determined, but also extracting estimates for them from experimental data is not straightforward, and these estimated values exhibit a wide range of variability. Furthermore, well-defined functional outputs do not typically exist, especially with regards to interneurons in the mammalian CNS [25]. Thus, in this paper we propose and use a cycling approach that encompasses ensemble modeling (Fig. 1) and is applied to a specific, quantitative question. While the use of ensembles of neuron models itself is not new, the difference and novelty here lies in our overall approach, with a key aspect being the design of the database of models to consider from the beginning. Specifically, it is not mainly or only about obtaining a limited set of optimal cellular models for subsequent use in network configurations [26], [27] but, rather, more about determining particular hypotheses, finding particular cellular-based predictions to consider experimentally, and evaluating what aspects in the model should be addressed and improved next. In this way, we can progress toward obtaining an understanding of the balanced contribution of the various conductances in spatially extended models, and to expose essential model limitations since our models are never biologically complete. Thus, with our approach, we aim to provide specific guidance for experimental work and a rationale for what makes the most sense to consider next. This supports the overall effort of determining essential balances of densities, types, kinetics and distributions of biophysical conductances that control dynamics in particular cell types for network functioning.

Our approach is described by a four-step cyclical process (Fig. 1). *Step 1* involves: (i) developing the base, reference model(s) from which a database of models will be derived, (ii) designing the database given the specific question being considered, and (iii) performing the multiple simulations given the determined database design and the experimental data protocols. *Step 2* involves: (i) building the databases for model and experimental comparisons and (ii) extracting appropriate models using some principled criterion. *Step 3* involves analyzing the good models to gain mechanistic insight into their function. Finally, *Step 4* involves: (i) examining the specific question considered in the database design, (ii) determining limitations that would subsequently update the reference models of *Step 1*, and (iii) suggesting and designing experiments for confirmation of the insights from *Step 3* as well as for further physiological investigation.

In the work here, we examined ion channel conductances and distributions of hippocampal O-LM hippocampus. We note that although we present and describe an overall cycling approach (Fig. 1), aspects of all steps of the cycling approach are not given in the present paper.

### Experimental data usage in developing and designing multi-compartment neuronal model databases

Experimental data was used as constraints for the model development (Fig. 1, Step 1(i)). The conductance densities of the voltage-gated ion channels in the model, the model's passive properties, and the morphologies of the model were all constrained using O-LM cell data where possible, building on previously developed multi-compartment O-LM cell models [22]–[24] (See Methods for full details). Then, using reference models as a base and with particular questions in mind to examine a neuron's character, a model database was designed (Fig. 1, Step 1(ii)). Here, we were interested in examining whether *I*_h_ could be present in dendrites and so distributions that included *I*_h_ in dendrites were considered in the database design. Although it has long been known that *I*_h_ exists in O-LM cells [19], its distribution along the cell's soma and dendrites is unknown, as compared to *I*_h_ in pyramidal cells that have a non-uniform dendritic distribution [28]. Two morphologies and a range of conductance densities and distributions were used. We chose to use a brute-force, or coarse grid database approach for our work both due to its simplicity in implementation and relatively comprehensive coverage of the conductance density parameter space [6], [8]. For each combination of model parameters, an instance of the multi-compartment O-LM model was simulated (Fig. 1, Step 1(iii)) on a high-performance computing cluster [29] by applying a current clamp protocol identical to that performed on O-LM cells in mice. The resulting model outputs – consisting of voltage traces recorded from the soma – were saved and processed on a local multicore computer running Ubuntu Linux.

### Application of a quantitative metric to rank the ensemble of O-LM models

**Figure 2:**
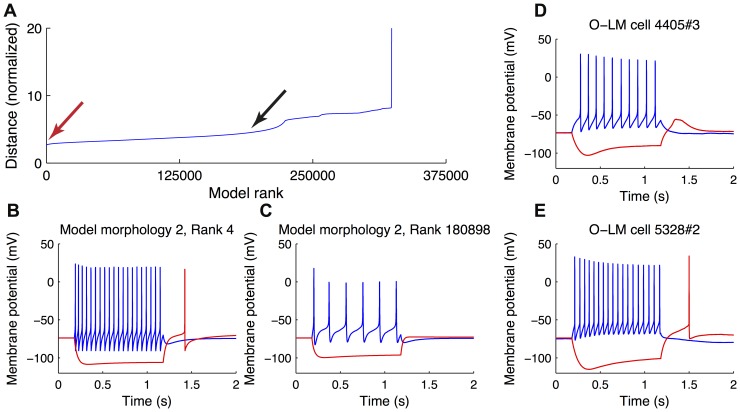
Ranking of O-LM models against experimental data. (A) The ranking of models against O-LM cell experimental recordings shows a gradual decrease of the goodness-of-fit of a given model as the rank of the model becomes poorer. Hyperpolarizing and depolarizing voltage responses of two representative models, a highly-ranked one (B, red arrow in A) and poorly-ranked one (C, black arrow in A) are shown for comparison purposes. Examples of experimental voltage traces are shown in (D, E).

We next processed raw voltage trace data from the total set of models simulated, and extracted quantitative metrics from these traces so as to enable direct comparisons between model and physiological O-LM cell output (Fig. 1, Step 2(i)). The goal of this step was to develop a principled way to determine which model outputs best corresponded to O-LM cell voltage outputs. Toward this goal, we used PANDORA, a MATLAB toolbox designed for the statistical analysis of model and experimental voltage trace data [30]. We imported both the model and O-LM cell experimental data into PANDORA and performed a ranking function on the model outputs, where each model was assigned a distance (an error measure) with respect to the entire experimental dataset (see Methods). As described in detail in the Methods, this measure is a quantifiable distance metric that is a statistical measure of all of the model's electrophysiological features compared with experimental ones and provides an objective test of its goodness-of-fit to the experimental data. We used equal weighing of the features to avoid the unintentional introduction of bias that would result by considering one or multiple features to be more important than others. We thought this reasonable to do at this time since it is currently unknown which features are critical for O-LM cell function in network and behavioural contexts. It is also important to note that this distance metric takes into account the biological variability as the various features are weighted by their standard deviation. The models were sorted according to their distance from the experimental dataset such that highly-ranked models had low distance values, and poorly-ranked models had high distance values (Fig. 2A). Voltage responses to ±90 pA current steps illustrated that a highly-ranked (Fig. 2A, red arrow) O-LM cell model (Fig. 2B) better represented O-LM cell properties than a poorly-ranked (Fig. 2A, black arrow) O-LM cell model (Fig. 2C), as confirmed by comparing several features, since the distance measure takes all features into consideration (see Methods). Examples can be viewed in Fig. 2 where highly-ranked models (Fig. 2B) better represented the empirical set of physiological O-LM cell recordings (Fig. 2D, E), as compared to lower-ranked ones (Fig. 2C). Thus, highly-ranked models seemed to capture important intrinsic properties of O-LM cells which the poorly-ranked models did not.

### Consideration of principled criteria for determining subsets of appropriate O-LM models in the ensemble

**Figure 3:**
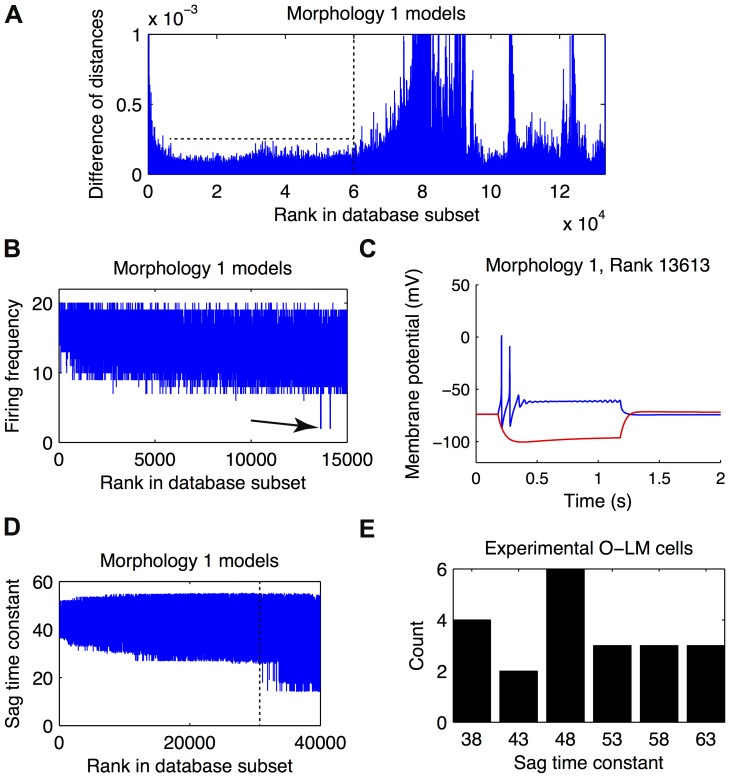
Extracting subsets of appropriate O-LM models from the database. (A) Plot showing the general subset cutoff determined by visual examination of the derivative of the distance metric with respect to the model ranking in the database. Vertical dashed line shows cutoff point. (B) The firing frequency plotted as a function of model rank demonstrates one restricted subset of O-LM models. The arrow points to the first failure-to-fire model, thus marking the cutoff point for this restricted subset. (C) The voltage traces of the failure-to-fire model shown in (B). (D) The time constant of the hyperpolarization-induced sag plotted as a function of model rank. The vertical dashed line shows the point in the ranking at which the time constant starts to deviate from the experimentally observed time constants. (E) Histogram of hyperpolarization-induced sag time constants within the experimental O-LM cell dataset.

Although highly-ranked models better represented O-LM cells than poorly-ranked models (Fig. 2), it is not obvious where a cut-off point should be applied to distinguish the two sets as the distance metric considers all features in a ranked fashion. A principled criterion is therefore needed to extract appropriate models (Fig. 1, Step 2(ii)). We first considered determining a cut-off point from the distance measure itself, noting that the distance measure incorporates consideration of a multitude of electrophysiological features (see Methods and Supplementary Materials). We refer to this cut-off point as the general criterion. This was done by plotting the slopes of the distance measure with respect to the model rank, which is equivalent to the difference of distances between adjacent models in the ranking. In order to test whether there were morphology-specific differences in the intrinsic dynamics of the O-LM models, the models using each of the two reconstructed O-LM cell morphologies were ranked and analyzed separately. After plotting the slopes for models of both morphologies, it was clear that the distance values changed rapidly in the first few thousand models, after which they increased at a relatively steady pace. In other words, model errors accumulated at a constant rate (Fig. 3A, horizontal dashed line). Eventually, the distance values of the ranked models for both morphologies started changing again at a more rapid rate. We therefore set the point at which the ranked models started to rapidly increase their distance values as the cut-off (Fig. 3A, vertical dashed line), as chosen by eye. For models of morphology 1, this resulted in the first 60,000 highly-ranked models counting as appropriate O-LM cell representations (Fig. 3A); likewise, for models of morphology 2, the first 90,000 highly-ranked models were incorporated into the ensemble of appropriate O-LM cell representations (not shown). This total set of 150,000 models was considered the general subset of appropriate O-LM models.

To assess the validity of the general criterion, we additionally considered a more restricted criterion in order to check whether the conductance density balances found in the two subsets would overlap. For this, we chose representative electrophysiological measures for both the depolarizing and hyperpolarizing current step voltage traces. We used the firing frequency of the models during the current injection step as a representative measure of depolarizing current step traces and the time constant of the hyperpolarization-induced sag response as a representative measure of hyperpolarizing current step traces (Fig. 3B–D). These specific measures contributed to the overall distance measure metric. When comparing the firing frequencies of the models for both morphologies, we noticed that early on in the ranking, some models exhibited behaviour which we termed failure-to-fire. These models were characterized by a combination of conductance densities that prevented the model cell from firing more than one or two action potentials during the +90 pA current injection step (Fig. 3C). Since none of the experimental O-LM cell voltage traces had an observed instance of this failure-to-fire behaviour, we deemed models that possessed this characteristic to be potentially inappropriate O-LM cell representations, and set the restricted cut-off at the rank prior to the first failure-to-fire model. In the case of models of morphology 1, the first failure-to-fire model occurred at rank 13,613 (Fig. 3B) whereas for models of morphology 2, the first failure-to-fire model was found at rank 19,245 (not shown). The resulting set of 32,856 models (13,612+19,244 models without failure-to-fire behaviour) was considered one candidate for a restricted subset of appropriate O-LM models as determined by the firing frequency measure. For the representative measure of the hyperpolarizing traces, the time constant of the sag response, we plotted the time constants as a function of model rank (Fig. 3D) and compared them to the time constants exhibited in the experimental dataset, by plotting the histogram of time constants for the latter (Fig. 3E). We observed that the time constants for the models of either morphology exhibited appropriate values for the first tens of thousands of models – as determined by being within the range observed in the physiological O-LM cells (Fig. 3E). However, at a certain point, the time constants became markedly lower and fell outside the range of those observed in the physiological O-LM cells (Fig. 3D, dashed line). We marked the approximate rank at which the models started exhibiting inappropriate time constants as another restricted cut-off point. This point was much further down the ranking of models than the restricted cut-off point for the firing frequency criterion, however (compare the location of arrow in Fig. 3B and dashed line in Fig. 3D; data for models of morphology 2 was similar and is not shown). Therefore, we only considered the cut-off point determined by analyzing the firing frequency behaviour, above, forming the restricted subset of appropriate O-LM models to be used as a comparison to the more general subset determined by the difference of distances metric.

### Three co-regulatory balances were found between conductances in the subsets of appropriate O-LM models in the ensemble

**Figure 4:**
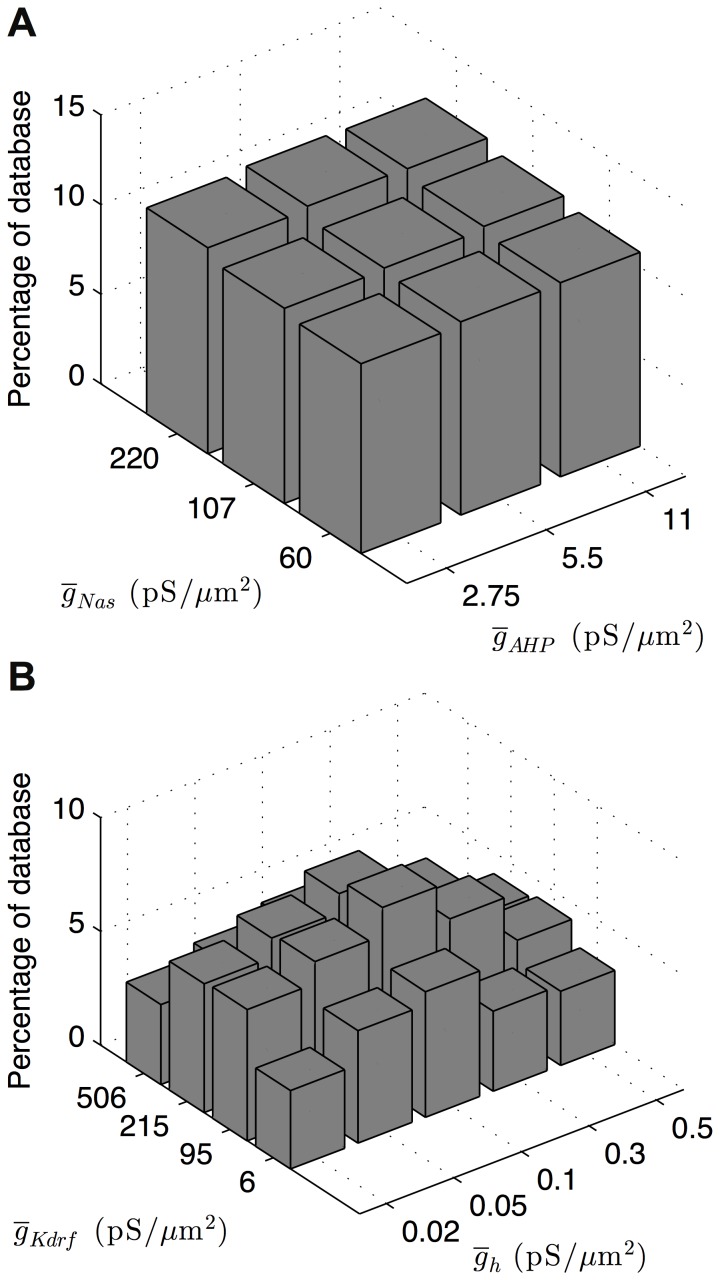
Conductance histogram plots showing no clear interaction or local preference. Pairwise conductance histogram plots show the number of highly ranked models, expressed as a percentage of the general subset of O-LM models, that possess the parameter values for the two given combination of ion channel maximum conductance densities shown on the x- and y-axes. (A) Conductance histogram plot for the somatic sodium conductance and AHP potassium conductance demonstrating no clear interaction. (B) Conductance histogram plot demonstrating a local preference between the fast delayed-rectifier potassium conductance and h conductance. Note the peak in the middle of the conductance density range for both conductances.

**Figure 5:**
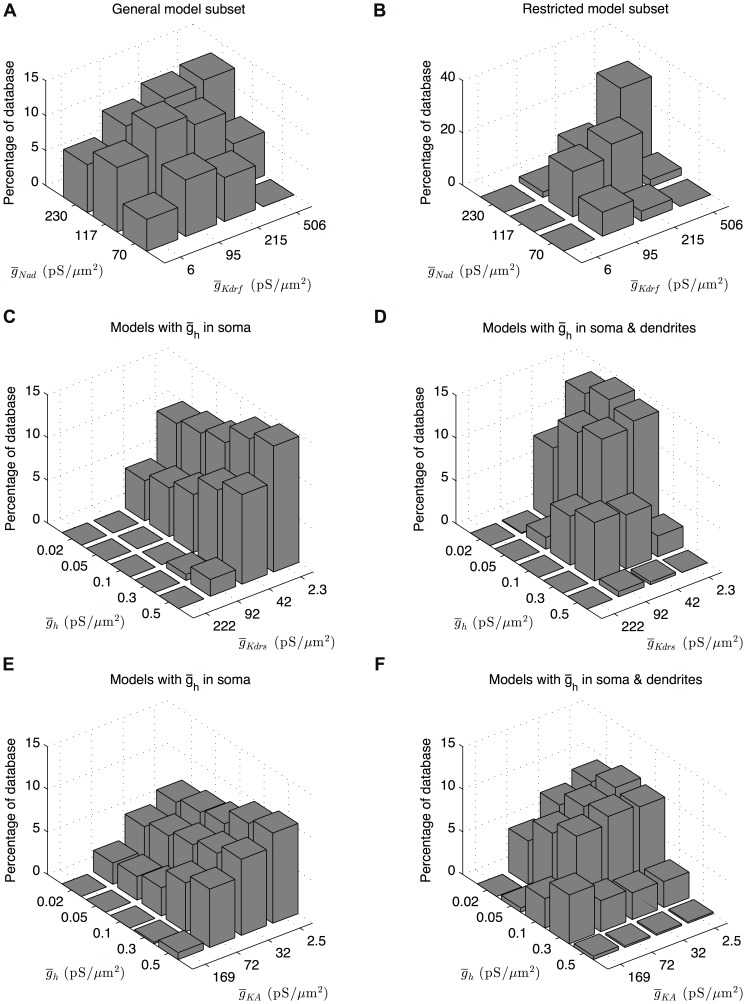
Conductance histogram plots for co-regulatory balances in the highly-ranked model database subsets. Histograms for pairwise conductance density values show three co-regulations between four of the nine active conductances present in the highly-ranked model subsets. Dendritic sodium is co-regulated with the fast delayed-rectifier potassium conductance as seen in both the general (A) and restricted (B) database subsets. The h conductance is co-regulated with both slow delayed-rectifier potassium conductance and A-type potassium conductance, as seen by the characteristic ridge in (D) and (F). These co-regulations are only present in the subset of models with h-current distributed uniformly across the somatic and dendritic compartments (D, F) and not the models with h-current distributed within the soma only (C, E).

Once the general and restricted subsets of appropriate O-LM models were determined, we examined the conductance density space of the models in each subset (Fig. 1, Step 3). For this, we constructed conductance histogram plots (Figs. 4, 5). These plots consist of histograms of the number of models contained in each subset of appropriate O-LM models that possessed any combination of conductance density values for the two ion channel conductances being considered. In order to avoid having to consider a very large set of conductance histogram plots, we used clutter-based dimension reordering (CBDR), an algorithm for the visualization of high-dimensional data in two dimensions, as a way to constrain which conductances we considered [31]–[33]. We considered the conductance density values for all the ion channel conductances of our models as the dimensions of the space, and the distance as the value of any given point in the space. The CBDR algorithm then determined an ordering of the conductances where the resulting structure of the distance space was most sensitive to the high-order conductances, and least sensitive to the low-order conductances. That is, changes in high-order conductance density values would result in large changes in the distance value for models, whereas changes in low-order conductance density values would not result in appreciable changes in the distance values. We could thus eliminate the low-order conductances from further consideration, as they did not seem to affect the behaviour of the models as exercised by the particular depolarizing and hyperpolarizing current injection step protocols used. The high-order conductances were *g*_Nad_, *g*_KA_, *g*_h_, *g*_Kdrf_, and *g*_Kdrs_, whereas the low-order conductances were *g*_Nas_, *g*_M_, *g*_AHP_, *g*_CaL_, and *g*_CaT_. We proceeded to compute the conductance histogram plots for all pairwise combinations of the high-order conductances as determined by CBDR analysis, in addition to one conductance that straddled the boundary between high- and low-order conductances, *g*_AHP_, that provided a check. The *g*_AHP_ conductance did not interact with any of the higher-order conductances, which served as confirmation that it, and all lower-order conductances, could be discounted from further analysis. We found three resulting categories of relationships between high-order conductances, similar to that found in previous work in an ensemble of model neurons of the crustacean stomatogastric ganglion network [34]. Using similar terminology, we found that conductances showed (1) no clear interaction, (2) a local peak or preference of conductance density values, or (3) a co-regulation. The first two cases were not deemed to be of interest in terms of uncovering putative conductance density balances. In the case of no clear interaction, any change in the maximum conductance density of one or the other conductance had no effect on the resulting models' goodness-of-fit as measured by the number of models contained within the general or restricted subsets of appropriate O-LM models and that possessed those conductance density values (Fig. 4A). For the second case of local preference, more models in the general or restricted subsets of appropriate O-LM models exhibited one particular combination of conductance density values, with tapering-off numbers of models exhibiting nearby combinations of conductance density values (Fig. 4B). In this case, although there was a clear preference for a particular value of one or both conductances, the two conductances did not interact in a meaningful way. The third category of relationships, that of co-ordinated regulation or co-regulatory balance, was demonstrated by the highly-ranked models exhibiting a distribution of pairwise conductances such that models with higher values of one conductance also had higher values of the other conductance. This is visually shown by a characteristic “ridge” in the conductance histogram plots of the two conductances in question (Fig. 5B,D,F). We elected not to do any formal statistical analysis of the co-regulations due to the sparseness of the data (3–5 maximal conductance considered) and also because the “ridge” can be clearly seen. Of all the examined pairwise combination of conductances, we only found three co-regulatory balances. Intriguingly, these three co-regulations were equally present in both the general as well as the restricted subsets of appropriate O-LM models. This indicated that the general subset, as determined by the general cut-off criterion using the difference of distance metric, was not too liberal in producing a set of models that best conformed to electrophysiological O-LM cell recordings. An example can be seen in Fig. 5A–B of two conductance histogram plots for the same two conductances, with one plot obtained from the general subset and the other from the restricted subset, and showing similar co-regulatory “ridges”.

The first co-regulatory balance observed was that between *g*_Kdrf_ and *g*_Nad_ in the case of all models of both morphologies (Fig. 5A–B). This indicates that the fast inward sodium conductance in dendrites is balanced against the fast outward delayed-rectifier potassium conductance: when maximum conductance densities of one of these conductances is increased or decreased, the maximum conductance densities of the other is also increased or decreased in a corresponding fashion in order to maintain physiological O-LM cell output. The remaining two co-regulations were only observed in those models that had *I*_h_ conductances distributed in their dendrites – that of *g*_h_ and *g*_Kdrs_ as well as *g*_h_ and *g*_KA_ (Fig. 5C–F). In this case, inward *I*_h_ conductance was co-regulated against both the slow outward delayed-rectifier potassium conductance as well as the outward A-type potassium conductance such that increases of *g*_h_ occurred with decreases of *g*_Kdrs_ or *g*_KA_. For models that expressed *I*_h_ in the somatic compartments only, no “ridge” (i.e., co-regulation) was seen between *g*_h_ and both *g*_Kdrs_ and *g*_KA_ (Fig. 5C, E). On the other hand, models with *I*_h_ uniformly distributed across all somatic and dendritic compartments exhibited these co-regulations (Fig. 5D, F). In all cases, there were no specific differences in the patterns of co-regulations found for morphology 1 and 2, suggesting that the co-regulations were not dependent on morphological details.

### *I*_h_ may be present in dendrites and a virtual protocol indicates that dendritic *I*_h_ may be detected experimentally

**Figure 6:**
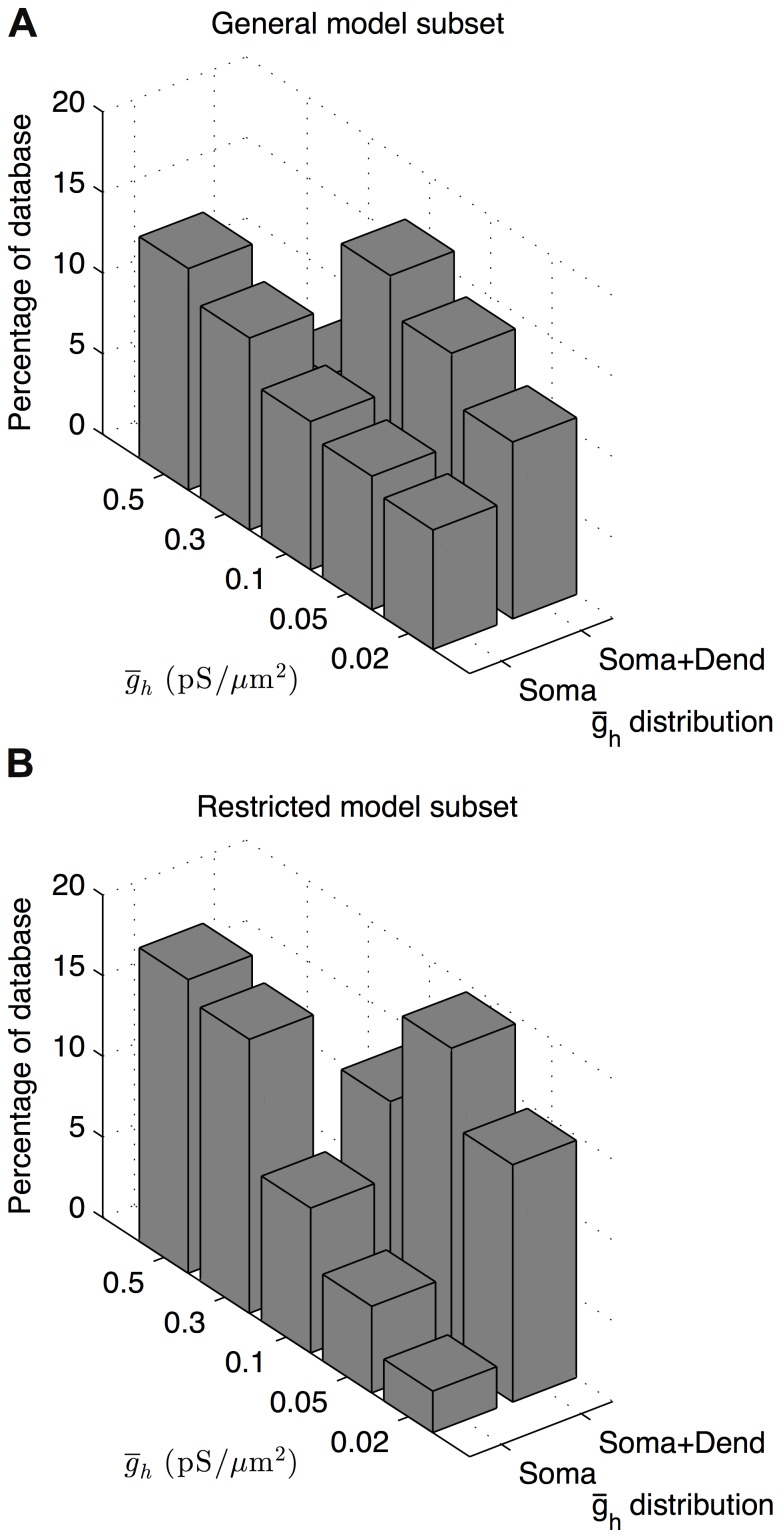
Inverse relationship between *I*_h_ maximum conductance densities and *I*_h_ distribution. Histograms for highly-ranked models in the general (A) and restricted (B) database subsets as a function of *I*_h_ distribution and *I*_h_ maximum conductance densities shows that highly-ranked models with somatic *I*_h_ only preferentially exhibited higher maximum conductance densities, whereas models with somatodendritic *I*_h_ preferentially exhibited lower maximum conductance densities.

Our database was designed to address the specific question of whether *I*_h_ is present on O-LM cell dendrites, which is unknown at present (Fig. 1, Step 4(i)). As a result, of particular interest to us was the finding that two co-regulations between conductances in the models depended on the presence of dendritic *I*_h_ as described in the previous section. We found that in both the general (Fig. 6A) and restricted (Fig. 6B) subsets of appropriate O-LM cell representations, somatic *I*_h_ only models preferentially expressed higher levels of maximum conductance densities of *I*_h_ relative to those with *I*_h_ conductances uniformly distributed in both soma and dendrites. This makes sense since one would expect that a wider distribution would not need as high a density to maintain overall balances. Furthermore, there were similar amounts of highly-ranked models in both subsets of appropriate O-LM models, regardless of model morphology, that had somatic *I*_h_ only (77,806 models in the general subset) as well as somatodendritic *I*_h_ (76,194 models in the general subset). The equal likelihood of models with either distribution of *I*_h_ channels being ranked highly in the ensemble of O-LM models, given that their *I*_h_ conductance densities were appropriately balanced, therefore did not allow for a clear prediction of dendritic *I*_h_ conductances in biological O-LM cells.

**Figure 7:**
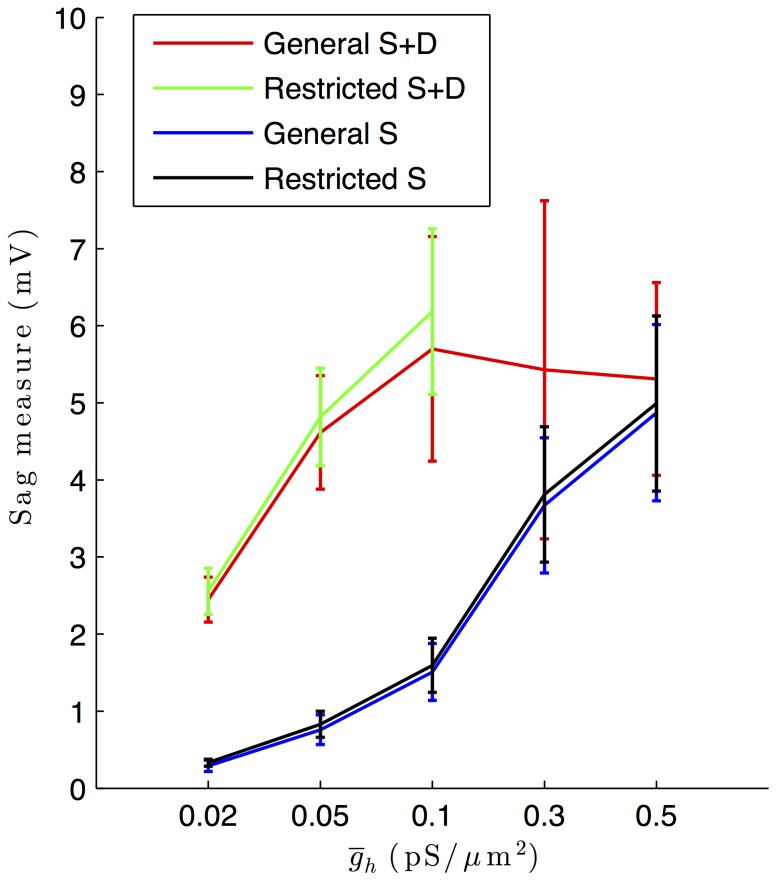
Hyperpolarization-induced sag response varies as a function of *I*_h_ maximum conductance density and somatodendritic distribution in models. Plots of the average sag response (mV) of model outputs as recorded in the soma as a function of different *I*_h_ maximum conductance density values across different model subsets. The general and restricted subsets of highly-ranked models with *I*_h_ in soma and dendrites are shown in red and green, respectively (“General S+D” and “Restricted S+D”, respectively, in the figure legend). The general and restricted subsets of highly-ranked models with *I*_h_ in soma only are shown in blue and black, respectively (“General S” and “Restricted S”, respectively, in the figure legend). Error bars denote standard deviations of the sag response in the respective model database subsets.

Close examination of our highly-ranked models revealed that they did not adequately capture the hyperpolarization-induced “sag” response typically observed in O-LM cell experimental data. This can be seen in Fig. 2, where the biological cells (Fig. 2D,E) show larger sag as compared with the highly-ranked models (e.g., Fig. 2A). Specifically, this measure, *PulsePotSag*, has a mean of 14.2 mV and a standard deviation of 3.1 mV in the experimental dataset (see Table S1), whereas its value maximally approaches 8 mV in any of the models. This is shown in Fig. 7 where this measure is plotted for general and restricted sets, and for models with or without dendritic *I*_h_. Considering the models without dendritic *I*_h_, we see that as the *I*_h_ conductance density increases, the sag amplitude also increases. One might think that if we simply increased this value more, the sag amplitudes in the models could better represent those seen in experiment. However, this is not the case, as an earlier version of our model database, with larger *I*_h_ conductances, exhibited unacceptable models (not shown) when the *I*_h_ maximal conductance densities were larger than 0.5 pS/µm^2^, which is the upper range of values in the current database. In other words, this increase in sag amplitude with increasing *I*_h_ conductance is due to a balanced increase of *I*_h_ conductance, as other conductances in the variety of models are not the same. Now, considering the models with dendritic *I*_h_, a larger sag amplitude can be obtained, but with the restricted set, models do not possess *I*_h_ maximum conductance densites greater than 0.1 pS/µm^2^ (Fig. 7, green line). Again, this increase in sag amplitude with larger *I*_h_ conductances is a balanced response. We note that these observations are a result of our database analyses, and would not have been feasible to uncover using hand-tuned modeling. We further note that our database design of models with and without dendritic *I*_h_ allowed us to easily examine what differences might exist between the two cases and to clearly show that dendritic *I*_h_ models are better at capturing the sag amplitude feature. However, as noted above, the sag amplitude feature is a clear limitation of the models (Fig. 1, Step 4(ii)).

**Figure 8:**
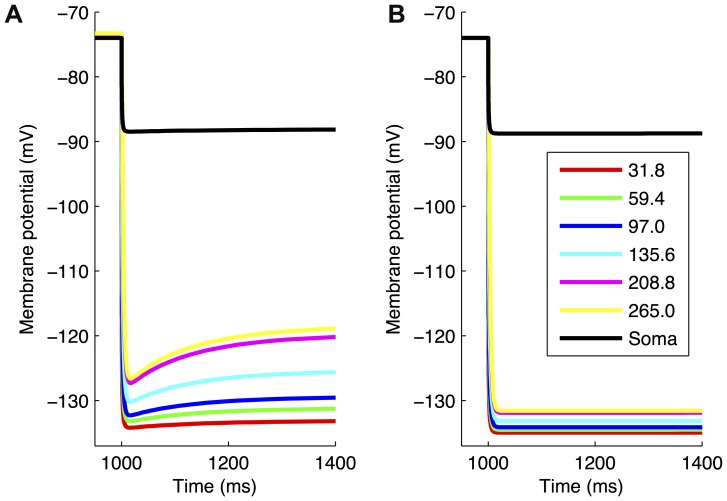
Virtual protocol on models indicates how dendritic *I*_h_ may be detected experimentally. Highly-ranked models were subjected to a mixed voltage/current clamp virtual experimental protocol as described in the main text. Somatic and dendritic traces for a highly-ranked model with *I*_h_ in soma and dendrites (A) and *I*_h_ in soma only (B). The maximum conductance density for *g*_h_ was 0.5 pS/µm^2^ for the model with *I*_h_ in soma and dendrites (A) and 0.3 pS/µm^2^ for the model with *I*_h_ in soma only (B). Both models used morphology 1. Somatic traces are shown in black, and dendritic voltage traces, measured along various points on the same dendritic branch, are shown at distances for both models as per the figure legend (all numbers in µm).

We next considered how one might assess whether dendritic *I*_h_ is present in O-LM cells, noting that we already know that *I*_h_ is present in O-LM cells [19], so it is not simply a matter of applying pharmacological blockers. We developed a virtual experimental protocol and applied it using highly-ranked models of both morphologies, *I*_h_ distributions, and high vs. low *I*_h_ conductance densities, that is, a total of eight different highly-ranked models. The protocol consisted of a mixed voltage clamp/current clamp virtual experimental protocol as follows: somata were held at a resting membrane potential of −74 mV using a voltage clamp while, simultaneously, a current clamp provided –5 nA (for morphology 1) or –2 nA (for morphology 2) hyperpolarizing tonic pulse in a proximal dendrite of the models 30 µm away from the soma. A distance of 30 µm was chosen as it was deemed to be far enough to consider for dendritic *I*_h_ presence, but not too far to be too difficult, given the extreme challenges in performing dendritic recordings on inhibitory cells. Applying this protocol to the eight highly-ranked models in our ensemble of different characteristics allowed us to evaluate the presence of dendritic *I*_h_. The somatic voltage responses did not demonstrate a sag both in cases of models with somatodendritic *I*_h_ (Fig. 8A, black trace) and somatic *I*_h_ only distributions (Fig. 8B, black trace). Only models with high *I*_h_ in dendrites showed any demonstrable sag (Fig. 8A, colored traces, with maximum *g*_h_ of 0.5 pS/µm^2^), except in the case when the dendritic *I*_h_ conductance was low (traces not shown). Furthermore, voltage measurements further away from the soma along the same dendritic branch showed less hyperpolarized steady-state values with more pronounced sag (Fig. 8A). On the other hand, lack of dendritic *I*_h_ consistently resulted in no sag response even with high *I*_h_ conductance (Fig. 8B, colored traces, with maximum *g*_h_ of 0.3 pS/µm^2^). Crucially, no models with somatic *I*_h_ only exhibited any sag response with the protocol, indicating that any somatic sag current effects do not propagate to dendrites. Thus, we conclude that for a sag to be measured in an O-LM cell using the mixed VC/IC setup as outlined above, *I*_h_ must be present in the dendrites at high enough densities. We note that these “high densities” are consistently lower than those measured in pyramidal cells [28].

## Discussion

### Summary and Predictions

We have proposed and used an approach in this work that is contingent on, and emphasizes the close inter-dependence of modeling and experiment for the model development, for the model database design and for providing specific predictions from the ensemble model analyses.

In the present study, we wished to investigate the question of whether hyperpolarization-activated inward currents, *I*_h_, (due to HCN channels) could be present on the dendrites of oriens-lacunosum moleculare (O-LM) inhibitory cells located in the CA1 region of hippocampus. This is currently unknown, and it is an important aspect to consider as dendritic *I*_h_ could have a strong impact on incoming synaptic input. Because it is difficult to determine the answer to this question by direct experimental approaches, we were motivated to develop a computational modeling approach that could address this question. As such, we developed a cyclical ensemble modeling approach (Fig. 1) for investigating the interaction of voltage-gated conductance densities and distributions that give rise to intrinsic, cellular output, and applied it to O-LM cells. By examining the pairwise interactions of maximum conductance densities for the eleven different voltage-gated ion channels present in each model of the experimentally-constrained resulting ensemble of O-LM models, treating somatic and dendritic sodium conductances as well as somatic and somatodendritic *I*_h_ separately, thus giving rise to 55 total possibilities, we found only three instances of co-regulatory balances between conductance densities. Two of these co-regulations involved the *I*_h_ current, suggesting that O-LM cell electrical activity may be sensitive to *I*_h_ conductances. The third is between the dendritic fast inward sodium conductance and the fast outward delayed-rectifier potassium conductance. Given that these two conductances have similar fast kinetics and dendritic distributions, but opposite polarities, it is perhaps not surprising that this positive co-regulation emerges from our analysis. We interpret this particular co-regulation observation as a validation of our ensemble modeling analysis. We also developed a mixed voltage/current clamp protocol and tested it on our highly-ranked models in order to test for the presence of dendritic *I*_h_, if performed experimentally.

From both the co-regulations found and the experimental protocol tested, we make three primary predictions regarding the physiological character of *I*_h_ currents in O-LM cells. The first is that the *I*_h_ conductance is smaller than that measured in CA1 pyramidal cells [28]. Our ensemble of models had 0.5 pS/µm^2^ as the largest allowable value for the maximum conductance density of *g*_h_. In a previous version of our ensemble, this was the lowest value, and any larger values produced universally inappropriate O-LM cell output (data not shown). Second, due to the observed co-regulations of *g*_h_ with both *g*_KA_ and *g*_KDRs_ in the case of models with uniform somatodendritic *g*_h_ conductance, if a positive correlation is observed between *g*_h_ and either of these two potassium conductances in O-LM cells, we predict that it is likely that *I*_h_ is present on those O-LM cell dendrites, as only the models in our ensemble with dendritic *I*_h_ exhibited these co-regulations. Third, using our mixed voltage/current clamp experimental protocol, we predict that sag will be observed in the dendritic compartment only when dendritic *I*_h_ is present; somatic *I*_h_ will not propagate into the dendrites. It is critical to note that this prediction arises because we had a set of appropriate O-LM models with differing parameter values from which to apply the protocol. With a single hand-tuned model, this would not have been possible. These findings and predictions point to the utility of the ensemble modeling approach as an investigative tool of a neuron's properties. We note the importance of determining the physiological question at the outset of the approach in order to design the appropriate model database to be built and analyzed for insights and predictions.

### Comparison to other ensemble modeling approaches

The approach taken in our work here can be thought of as a combination of previous ensemble modeling studies such as that of Günay and colleagues [35] as well as Hay and colleagues [26]. Günay and colleagues built model databases to show that they could capture the variability seen in experimental recordings from globus pallidus neurons. Their results suggested increased attention on dendritic fast sodium conductance characteristics that were examined and proposed to have functional importance in Parkinson's disease [36], [37]. The focus of this study, therefore, was on the potential functional role of particular cell types and ion channels, with the network contributions of globus pallidus neurons in this case. On the other hand, sophisticated and comprehensive approaches utilizing evolutionary algorithms, a class of optimization algorithm that includes multiple objective optimization (MOO) [26], [27], have been developed to obtain populations of models that represent the variable experimental data. Hay and colleagues applied a MOO approach to layer 5b (L5b) pyramidal cells in order to capture their experimental variability, as well as to maintain a limited set of output characteristics identified *a priori*. This included both perisomatic voltage-gated Na^+^ and dendritic Ca^2+^-generated action potentials. These models were designed and constrained in order to broadly capture interesting experimental features of L5b pyramidal cells and therefore used as building blocks large-scale modeling studies of cortical networks.

Since one never has the full extent of experimental data to fully constrain multi-compartment models, along with the fact that models will never fully encompass all biological details, our focus here is on approaching the problem of considering specific questions using a quantitative ensemble model approach. In the work here, we mean the intrinsic behaviour of O-LM cells and their role in hippocampal networks. In practice, this means having back and forth considerations between model and experiment (i.e., the cycle schematic in Fig. 1), so that functional aspects and potentially important characteristics, for example, *I*_h_ in dendrites, can be examined. Thus, while our approach overlaps in some respects with other brute-force ensemble modeling techniques as per Günay and colleagues [35] as well as MOO approaches such as Hay and colleagues [26], our methodology differs – we design our ensemble databases to explore a specific question involving distributed biophysical conductances (unlike Günay and colleagues), and we use brute-force methods and a ranked distance metric (unlike Hay and colleagues) to build and evaluate our ensemble models.

Although sophisticated evolutionary algorithms of which MOO is an example are something that we could consider taking advantage of, these have a slightly different emphasis from our approach in that they inherently focus on model optimization. Because evolutionary algorithms can find optimal solutions more quickly than brute-force approaches, they are often considered to be more computationally efficient. However, in our approach we are interested in finding comprehensive subsets of appropriate solutions (models) from the entire parameter space. This becomes a more pressing issue as distributions of channels, which are not typically known, also need to be considered. Because evolutionary algorithms are subject to terminating at local minima, this means that in order for the algorithm to cover the entire parameter space reliably, many repeated runs are required, thus blurring the line between evolutionary algorithm and brute-force methods. Furthermore, a brute-force grid search may be more appropriate when a “complete” multi-compartment model in terms of biophysical ion channels is lacking. In other words, an approach that can comprehensively provide guidance as to the appropriate regions of parameter space, even if it initially covers a coarse grid of parameters, may be more desirable when considering the exploration of functionally relevant aspects of models. However, evolutionary algorithm approaches could be useful in examining more specific regions of the parameter space that are of interest. The complementary use of coarse-grid and evolutionary algorithm ensemble approaches should be considered in the future.

Using brute-force database techniques as a form of model analysis was proposed by Prinz and colleagues [38], expanding on the work in [39], where the point was made that analytical techniques such as bifurcation analysis are not possible when the systems under consideration, such as multi-compartment computational models, possess a large number of variables with overlapping time scales. It may be that quantitative analysis of large numbers of simulations could be used together with simplified models that can be analyzed using bifurcation theory, by linking particular balances and mechanisms in multi-compartment and simplified models respectively. In a similar vein, Achard and DeSchutter [40] showed that different parameter sets of a cerebellar Purkinje neuron model could produce similar output, and described the examination of the model's parameter landscape as an important consideration for understanding neuronal homeostasis. Both brute-force and genetic algorithm approaches to model parameter space explorations are summarized in [41]. There, however, the emphasis was on model optimization, that is, finding the subsets of models that best fit the experimental data, as opposed to the approach described in this work of thoroughly examining the model space to find patterns in parameters that both underlie good models as well as determine when and why models fail to reproduce experimental data.

In an excellent perspective, Marder and Taylor [8] described why populations of models should be built, and provided a summary of experimental and computational work describing this paradigm shift in the field. We note that these methodological advances have been largely spearheaded by work in the crustacean stomatogastric ganglion network (STG), where there are not only detailed cellular models – to build the model ensembles – but also deep insights into network function and behavioural contexts that can be uniquely leveraged to confront and examine issues of variability and homeostasis. Hudson and Prinz [42] took advantage of their original database [38] in order to examine the robustness of activities and linear correlations. In that work, they found that *I*_h_ and *I*_A_ were robustly correlated in their model database. Interestingly, *I*_A_ and *I*_h_ co-regulations were also found in the present ensemble database of appropriate O-LM models. However, Hudson and Prinz's work used single compartment models, whereas we found co-regulations of *I*_A_ and *I*_h_ only when *I*_h_ was distributed in both soma and dendrites.

Prinz and colleagues [43], additionally reviewed in [44], examined how tightly regulated parameters needed to be for network output and found that there was considerable flexibility in parameters required for appropriate model output, indicating that there could be significant animal-to-animal variability in neuronal ion channel densities. Although it is possible to simplify the complexity of models in order to perform dynamical systems analysis [45], there are always limitations in model details that make analysis difficult. The simple motor pattern system of the STG allowed these questions to at least be addressed in a behaviourally-relevant way.

It is important to note that most multi-compartmental models are not developed with biophysical details from experiment. This is more prevalent in vertebrate systems, especially inhibitory cells, which are only beginning to be characterized. Hippocampal interneurons are an example of cell types whose network roles are not well understood in terms of computational principles [46], despite the plethora of experimental data and theories regarding interneuron function (e.g., [11], [20], [25], [47], [48]). This is therefore another reason to support the use of coarse-grid or brute-force parameter search techniques such as the one used in this work, since a more inclusive examination of models is needed when the functional role, and importance of the model's various ion channels for fulfilling that role, are unclear. This is in line with critiques of the database approach by Nowotny and colleagues [45], where they surmised that an important function of such database approaches may be to determine general principles of model behaviour in the parameter space, rather than uncover all possible dynamical regimes. Furthermore, as the models are used to examine ideas regarding cell function, and experimental predictions are formed and verified, there will need to be several cycles of such work, where further ensemble modeling studies are done before a more complete understanding of interneuron function can be developed.

### The role of O-LM cells and the relevance of *I*_h_ currents

As techniques grow more sophisticated, we learn more about the cell-type specificity of intrinsic and synaptic characteristics of interneurons [49], along with their specific, putative roles [12], [13]. The precise roles that interneurons perform during physiological functioning of hippocampus are not known. For O-LM cells, it was previously proposed that, due to their h-current kinetics, O-LM cells could play a key role in generating theta-frequency rhythms in the CA1 region of hippocampus [20]. This line of thinking originated with the observation that O-LM cells fire intrinsically at approximately theta frequencies in the absence of synaptic input [19]. Subsequent experimental work using dynamic clamp techniques, however, provided evidence against this idea [21]. Another putative role for O-LM cells in the CA1 hippocampal microcircuit emerged in studies of feedback excitation of O-LM cells by pyramidal neurons [50], [51]. These studies showed that CA3 (via Schaffer collaterals) activation of CA1 pyramidal cells excited O-LM interneurons, which would then, in turn, inhibit entorhinal cortical input at the distal dendritic regions of pyramidal cells. Conversely, therefore, decreased Schaffer collateral activation of pyramidal cells resulted in increased responses to entorhinal cortical input. This alternation of input strength between entorhinal cortical regions, which encode sensory information, and CA3, representing previously stored associations, onto the CA1 network, has been proposed as a way for CA1 to support memory encoding and retrieval processes [51] or to function as a match/mismatch detector between CA3 stored representations and entorhinal cortical sensory information [52]. More recently, a computational modeling study suggested that encoding and retrieval dynamics in CA1 occurs as a result of differential interneuronal activation across phases of the theta cycle [53]. In particular, O-LM cells were suggested to serve as “gates” of information flow onto pyramidal cells, switching between novel sensory information from entorhinal cortex and previously stored information from the CA3 region. A recent optogenetic study of O-LM cells provided some confirmation for this hypothesis in that a “gating” role for O-LM cells was indeed observed, but found to be dependent on cholinergic neuromodulation of O-LM cells, not based on theta frequency-dependent activation of O-LM cells [14]. Thus, the role of O-LM cells in hippocampal functioning is far from clear, but particular details that are contributing to our understanding are being uncovered. Together with insights derived from modeling studies, specific balances and mechanisms that are functionally important can emerge.

The voltage dependence and kinetics of *I*_h_ endow it with the possibility of contributing in many ways to cellular output - such as setting resting membrane potential and providing protective effect along with pacemaking possibilities, in general [54]. While we do know that *I*_h_ exists in O-LM cells [16], [19], and our ensemble modeling work here supports the possibility that *I*_h_ could be present in dendrites, how *I*_h_ might contribute to putative O-LM cell roles remains to be determined. A recent study using whole cell recordings on CA1 pyramidal neurons demonstrated the importance of particular *I*_h_ distributions in dendrites for allowing temporally coincident inputs from spatially distinct synaptic contacts to synchronously affect the soma [55]. Given this importance of *I*_h_ in compensating for delays in signals from spatially spread-out complex dendritic arbors in pyramidal cells, it may be the case that similar mechanisms are at play in O-LM cell dendrites, if *I*_h_ is indeed present there. More generally, such work exemplifies the critical contributions of active conductances in dendrites for neuronal synaptic integration [56]. O-LM neurons are the targets of diverse sources of synaptic input, both from excitatory recurrent collaterals from pyramidal cells as well as excitatory cholinergic afferents and inhibitory septohippocampal GABAergic projections [50], [57]–[59]. Cholinergic activation of hippocampal interneurons in particular has been shown to be cell type-specific in its neuromodulatory effects by engaging interneurons through a variety of post-synaptic receptors, giving rise to differential effects on interneuron contributions to network oscillations [60]. Thus, given the varied incoming synaptic inputs to O-LM cells, it is important to know whether *I*_h_ is present in the dendrites of O-LM cells as well as how it is distributed.

### Limitations and next steps

A limitation of our approach pertains to the nature of the distance metric. Although we incorporated 103 total electrophysiological measures in the standard score, or z-score, normalized distance statistic when comparing model to experimental traces (see Methods for details), we used a liberal approach of including more measures than were necessary; that is, some redundancies in the measures exist. Furthermore, all measures were equally weighted; that is, no one measure was considered more important than any other for determining the ranking for a given model against the experimental dataset. We settled on this approach in order to develop a general technique of matching models to experimental data without needing to arbitrarily weigh certain measures as more or less important than others, which might have biased the resulting ranking. As a result, some models were ranked highly that were not appropriate O-LM cell representations, such as the “failure-to-fire” models shown in the Results section (Fig. 3C). Therefore, the distance metric we used may not be appropriate in all experimental and modeling contexts, and specific tuning may be required. In the case of the failure-to-fire models, a higher weighting of the *PulseSpikes* measure, which counts the number of spikes during the current injection period (Table S2), would have ensured that the failure-to-fire models were more heavily penalized, as their dearth of spikes would have led to a low *PulseSpikes* measure relative to the experimental dataset. However, such manual tuning of the distance metric is not desirable in general as there is no guarantee that all highly-ranked models that are in fact poor representations of experimental cell behaviour can be found. Additionally, without having a clear functional relevance of any given electrophysiological measure it would be unclear how to rationalize an increased or decreased weighting, so that weighting choices would be arbitrary. One way of avoiding the trap of manual adjustment is to weigh any measure that is more than, say, two standard deviations of the experimental dataset away from the mean more heavily than those below two standard deviations. This would help ensure that poor measures result in higher distance values for the models exhibiting such measures so that they would more likely to be lower-ranked. Alternatively, one could simply remove all models from the database that are not within the two standard deviations of the measure. Whether this results in an overall better set of models that are highly-ranked remains to be determined. We note that this alternative corresponds to that used by Hay and colleagues [26] in which a defined set of measures was chosen and evolutionary algorithms used to obtain models that exhibited measures that were within a couple of standard deviations of the experimental ones. However, this alternative is potentially more restrictive of the chosen set of ‘good models' depending on how many standard deviations one chose to use. In the approach taken here, the restriction pertains to the chosen cut-off value, and could encompass a range of standard deviations of the various measures. This general issue can be explored in future work.

A deficiency of our highly-ranked O-LM models is that other channel types, such as the calcium-dependent non-selective cation current (*I*_CAT_) [57] that could be present in O-LM cells are not included. However, this could be examined by performing another cycle (Fig. 1), and it would be interesting to know if similar co-regulations end up being present as those found in the present work. Furthermore, we designed our database to focus on *I*_h_ dendrites, noting that experimental evidence for other K^+^ channels and Na^+^ in dendrites was already known unlike *I*_h_. Given that our work supports the possibility of dendritic *I*_h_, we believe that an important next step is to consider non-uniform distributions, especially since non-uniform distributions of *I*_h_ are known to be present in other cell types such as pyramidal cells [28]. This is because we found that highly-ranked models with *I*_h_ in soma only as well as soma and dendrites were not able to exhibit sag characteristics as pronounced as those in experiment (e.g., see model and experimental traces in Fig. 2, and Fig. 7). This reveals the possibility of deficiencies in the model of *I*_h_ itself. Therefore, not only should non-uniform distributions of *I*_h_ be considered in future work, but also a careful examination of the mathematical formulation of *I*_h_, and consideration of variations in sag time constants.

A recent study has shown that CA1 O-LM interneurons comprise two functionally distinct subpopulations, one expressing 5-HT_3A_ receptors and exhibiting increased participation in kainate-induced oscillations as well as recruitment through serotonergic activation, and one lacking this receptor type [61]. The morphological and electrophysiological properties remained mostly uniform across the two O-LM subtypes, with the possible exception of slightly higher input resistance in the 5-HT_3A_ receptor-expressing O-LM cells. Nevertheless, the different developmental paths, with the 5-HT_3A_R-containing cells being derived from caudal ganglionic eminence and the non-5-HT_3A_R-containing cells being derived from medial ganglionic eminence, in addition to their differential recruitment via serotonergic drive and kainate-induced gamma oscillations, suggests that there may be separate functional roles of these two O-LM subtypes in CA1. The experimental data used in our present modelling work did not make distinctions between these subtypes as it did not include synaptic drives. A natural next step would be to include synaptic drives, embedding O-LM cells in network contexts, incorporating neuromodulation, and using *in vivo* O-LM cell data. This would then allow consideration of such O-LM subtypes, as well as contributions of various intrinsic O-LM properties in network functioning.

Experimental work in STG neurons has not only demonstrated co-regulations between different conductances [7], but also that correlations in ion channel mRNA expression levels do not always match with correlations observed in conductance densities of the channels [62]. This suggests that the electrical output of neurons might not actually require some of the co-regulations determined using ensemble modeling analysis, complicating the picture of ensemble modeling studies. Finally, there are indications that neuromodulatory influences may be more important than conductance correlations [63], [64]. As such, one could consider building-in homeostatic rules for co-regulation analysis of our O-LM interneuron models, as per [65], in order to examine the difference in co-regulations of resulting channel conductances there as opposed to varying conductance densities directly. However, this is probably premature to pursue for O-LM cells as it is unclear at present what typical cell output is required for network functioning, since we are dealing with a more complex and less well-characterized cell architecture and network functioning situation than STG neurons and networks.

Another consideration involves the input stimuli that should be used experimentally and in subsequent generation of the model databases. Ideally, stimuli should be such that they reveal the full dynamics of the nonlinear model or biological system, but what is most appropriate is unknown. Interestingly, work by Druckmann and colleagues [66] showed that noisy stimuli were not as effective as current steps and ramps were in constraining models. In this work we used two constant current injection steps consisting of –90 pA and +90 pA current steps, but a more systematic determination of the number and types of current injection steps or ramps should be considered in future iterations of O-LM ensemble modeling cycles. Also, in using a cut-off determined by eye (the general criterion), to obtain a subset of appropriate O-LM models, one could argue that this was arbitrary. However, the robustness of the co-regulations with both general and restricted cut-off criteria indicates that it is not critical to determine an exact cut-off value. Furthermore, if no cut-off was done – i.e., the entire database including clearly inappropriate models was used – then co-regulations as described here did not emerge when conductance histogram plots were examined (not shown). Also, due to the sparse sampling and the developing methodology, we elected to not perform statistical tests at this time, but instead relied on visual examinations of our conductance histogram plots.

In conclusion, we have proposed a novel ensemble modeling methodology, and used it with O-LM model cells and experimental data to gain insights. We expect that a continual cycling of our approach will help us determine essential biophysical conductances and balances for cellular contributions to network functioning for O-LM cells, as well as any other neuronal cell type for which a starting multi-compartment model has been developed, and a specific physiological question exists.

## Methods

### Experimental data

#### Hippocampal slice preparation

The experimental data used in the present work was acquired as part of a previous study [57]. All experiments were conducted in accordance with animal protocols approved by the National Institutes of Health (Animal Study Proposal #08-045). Mice (CF1 x 129J1) aged 14–21 days were deeply anesthetized by isoflurane volatile inhalation and sacrificed, with all efforts made to minimize suffering. The brain was rapidly removed and placed in ice-cold artificial cerebrospinal fluid (ACSF) with the following composition (mM): 87 NaCl, 2.5 KCl, 1.25 NaH_2_PO_4_, 25 NaHCO_3_, 25 glucose, 75 sucrose, 7 MgCl_2_, 0.5 CaCl_2_ saturated with 95% O_2_ and 5% CO_2_, pH 7.4. Transverse hippocampal slices of 300 µm thickness were cut using a VT1000S (Leica Microsystems, Bannockburn, IL, USA) or Vibratome 3000 Deluxe (Vibratome, St. Louis, MO, USA) and placed in a continuously oxygenated, warm (36°C) ACSF bath for at least 30 min before use.

#### Electrophysiological recordings

The set of recordings used in the present work consisted of those obtained during whole cell current clamp conditions of O-LM cells. The cells were maintained at approximately −60 mV, which resulted in a membrane potential of approximately −73.8 mV after a junction potential correction of −13.8 mV. To maintain the cells at −60 mV, a small negative bias or holding current applied through the somatic recording pipette (−8.0±4.0 pA, n = 11). Injection of a −5 pA current applied similarly would periodically be done in order to verify a tight seal with the cell membrane. Depending on experimental protocol, an additional depolarizing (+90 pA) or hyperpolarizing (−90 pA) would then be applied for a 1 s duration, after which the additional depolarizing or hyperpolarizing current injection would cease. The depolarizing current stimulus would result in regular action potential firing in the observed O-LM cells, with a “saw-tooth” [67] firing profile typically seen in O-LM cells and a long-lasting afterhyperpolarization. The hyperpolarizing current stimulus results in a characteristic “sag” back towards the resting potential, attributable to the presence of the hyperpolarization-activated current, *I*_h_. The experimental recordings used in this model database work consisted of ten identified O-LM cells in total, with traces including ±90 pA current steps chosen for each cell, resulting in a dataset of 56 total experimental traces.

### Reference multi-compartment model

#### Morphologies and passive properties

The reference models used in this work were adapted from previous multi-compartment models of O-LM cells, developed in conjunction with experimental data [23]. Because the current work included experimental data from another set of recorded O-LM cells [57], we refitted the passive membrane properties in the model to reproduce the transient membrane responses observed in the current set of experimental recordings. This was done by averaging 50 voltage-clamp seal tests from each O-LM cell, corresponding to the capacitative current due to a −5 mV step away from a −74 mV holding potential. The resulting passive properties for each morphology are specified in Table 1.

**Table 1:** Fitted passive properties for the two O-LM model morphologies.

Passive property	Model morphology 1	Model morphology 2
*R*_a_ (Ω ⋅ cm)	300	300
*C*_m_ ( F/cm^2^)	0.96857	0.9
*R*_m_ (Ω ⋅ cm^2^)	59,156	39,038
*E*_L_ (mV)	−73.588	−73.8424
*g*_KL_ (S/cm^2^)	9.9005×10^−10^	1.0015×10^−9^

#### Compartmentalization

For each of the two morphological reconstructions used for the models in this work, an appropriate number of compartments needed to be determined in order to maintain the spatiotemporal accuracy of simulations. The number of compartments was set using the fraction of the frequency-dependent length constant at 100 Hz, or λ_100_ [68], and was determined by setting up the “rig” in the NEURON simulation environment for fitting the passive properties of one model and morphology to experimental O-LM seal test recordings. An initial fraction of λ_100_ was assigned as the parameter to determine the number of segments within each section, and therefore the total number of compartments in the model. A trial run was then initiated in the Multiple Run Fitter (MRF) to determine the error of the model's membrane response to a −5 mV voltage clamp step compared to the experimental O-LM cell average. The passive properties were held fixed at the values defined in the reference model. Afterwards, the fraction of λ_100_ was lowered, resulting in a model with more compartments and hence greater simulation accuracy, and the MRF trial was re-run. This was continued until the error value for the model did not change appreciably, thus indicating that the model output was being simulated with sufficient accuracy. The λ_100_ fractions determined for the model morphologies 1 and 2 were, respectively, 0.0101 and 0.00465, resulting in 1,291 compartments for the former, and 2,413 for the latter. The resulting input resistance for the respective model morphologies were 474 MΩ and and 530 MΩ, with membrane time constants of 57ms and 66ms, respectively, for model morphologies 1 and 2.

#### Ion channel conductances

The model included nine active voltage-gated ionic conductances known to be present in O-LM cells. These are the sodium current as described by Hodgkin and Huxley, *I*_Na_, fast and slow delayed rectifier potassium currents, *I*_KDRf_ and *I*_KDRs_, respectively, the transient or A-type potassium current, *I*_A_, the L- and T-type calcium currents, *I*_CaL_ and *I*_CaT_, respectively, the calcium-activated potassium current, *I*_AHP_, the hyperpolarization-activated mixed cation current, *I*_h_, and the Kv7/KCNQ/M current, *I*_M_ [23], [24]. The mathematical expression of the sum of these currents' effects on the membrane potential is described by *I*_ionic_ is shown in Equation 1:
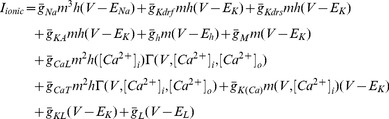
(1)


where *E*_Na_  = 50 mV, *E*_K_  = −95 mV, and *E*_h_  = −32.9 mV are the reversal potentials for, respectively, sodium, potassium, and the hyperpolarization-activated mixed cation channel. The kinetic equations for the ionic currents are as in [23]. The distributions of the conductances are described in Table 2. The distribution for *I*_h_ was set to uniformly spread over either all somatic compartments, or all somatodendritic compartments.

**Table 2:** Summary of maximum conductance density values used in the model database construction.

Active conductances	Maximum conductance density values (pS/ m^2^)	Compartmental locations	References
*g*_Na,s_ (somatic)	60, 107, 220	Soma	[69], [71]
*g*_Na,d_ (dendritic)	70, 117, 230	Dendrites, axon	[69], [70]
*g*_KDRf_	6, 95, 215, 506	Soma, dendrites, axon	[24], [69], [71]
*g*_KDRs_	2.3, 42, 92, 222	Soma, dendrites, axon	[24], [69], [71]
*g*_A_	2.5, 32, 72, 169	Soma, dendrites	[23], [24], [69], [70]
*g*_h_ (version 1)	0.5, 16, 53, 90	Soma only or soma and dendrites	[22], [28], [69]–[73]
*g*_h_ (version 2)	0.02, 0.05, 0.1, 0.3, 0.5	Soma only or soma and dendrites	[22], [28], [69]–[73]
*g*_CaL_	12.5, 25, 30	Dendrites	[24], [74], [75]
*g*_CaT_	1.25, 2.5, 5	Dendrites	[24], [74], [75]
*g*_AHP_	2.75, 5.5, 11	Dendrites	[24]
*g*_M_	0.375, 0.75, 1.5	Soma, dendrites	[23]

The third column shows the model compartments where the conductance in any given row was distributed. In all cases, distributions were uniform across the specified compartments. This yields 233,280 combinations of parameters.

#### Simulation and model output analysis

Selection of active conductance density ranges for database simulations. For the ensemble modeling approach, the maximum conductance densities were varied for the model, thus constituting a methodology for the models to exhibit different ion channel expression levels. The values that the maximum conductance densities for the various ion channels were allowed to take were determined on a case-by-case basis depending on what was previously known about that ion channel type, and specifically about its presence and somatodendritic densities in the O-LM cell. Table 2 lists the final maximum conductance density values used in the model database construction and references.

#### SciNet framework for running simulations

The ensemble modeling approach used here is the variant sometimes referred to as the brute-force approach because it depends on systematically varying all of the parameters and generating model output for each possible combination of parameters [41]. By varying the maximum conductance densities of the O-LM model in this work, of which there are ten (treating the somatic and dendritic sodium conductances separately – see Table 2), as well as the distribution of *I*_h_ maximum conductance density along somatic only versus somatodendritic compartments (2 options) and, finally, the morphology of the model used (2 options), there are a total of 933,120 possible models. Considering that experiments for both -90 pA and +90 pA current injections need to be applied to each model, this results in 1,866,240 total simulations that potentially need to be evaluated. Therefore, the use of high-performance computing (HPC) was required. For this work, the SciNet HPC supercomputer cluster was used for evaluating the model outputs. The SciNet General Purpose Computing (GPC) cluster consists of 3,780 nodes with 8 cores each [29]. Being able to handle all of the model simulations required significant automation. We implemented three tools in order to meet this criterion: (1) a script to generate the command-line invocations of all of the models; (2) fully automated NEURON code to evaluate the output of each; (3) an efficient system for finding missing models.

#### Fitting procedure for bias current

The membrane potential of each O-LM cell was held at a fixed voltage to ensure consistency in the state of the voltage-gated ion channels present in the membrane relative to action potential threshold. This was accomplished by dynamically varying the amount of bias current, or holding current which was injected prior to, and concurrently with, the subsequent ±90 pA hyperpolarizing or depolarizing current injection step in order to maintain a *V*_m_ of approximately −74 mV prior to the current injection step. However, many models would either exhibit premature action potential firing before reaching an experimentally appropriate bias current, whereas others would need too much positive bias current, relative to experimental values, to drive them to fire. Since these models did not contain appropriate O-LM cell characteristics, they were discarded. By following this procedure, 609,143 out of a total of 933,120 models were found to be inadequate, with 323,977 models being considered acceptable and retained for further analysis of conductance density balances.

#### Distance measures and model ranking

Once model outputs were obtained, they were imported into PANDORA's Toolbox, a MATLAB toolbox for the statistical analysis of experimental and model voltage traces [30]. We chose 11 electrophysiological measures for the −90 pA and 92 for the +90 pA experimental current clamp traces (see Tables S1 and S2). To obtain an aggregate measure of the “closeness” or error between a model and experimental trace, we used a ranking function provided by PANDORA that calculated the normalized Euclidean distance between all the models in the provided model database and the single experimental trace, as per Equation 2 [30]:
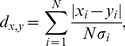
(2)where *x*_i_ and *y*_i_ represent the *i*th measure, out of *N* total measures, of the model and experimental traces, respectively, σ*_i_* is the standard deviation of the measure in the experimental database, and *d*_x,y_ is the resuting normalized Euclidean distance between model trace *x* and experimental trace *y*. The importance of the σ*_i_* normalization term is to penalize models whose measures differ significantly when those measures are tightly constrained in the experimental database – that is, when σ*_i_* is small. On the other hand, models with measures that vary significantly in the experimental database – that is, when σ*_i_* is large – will not be penalized by the distance calculation if they differ significantly from the experimental measure, *y*_i_. Effectively, the equation calculates the standard score, or z-score, of all of the model's measures – equally weighed – against the experimental measures. The resulting distance value, *d*_x,y_, represents how close of a match a model trace is to an experimental trace. Larger *d*_x,y_ values correspond to models that are “further away” from the experimental trace whereas lower *d*_x,y_ values correspond to models that are “closer”, or better matches with the experimental trace. Note that this corresponds to the distance between single model and experimental traces. Distance values of all model traces against all experimental traces were then summed and normalized by the number of experimental traces (Equation 3):
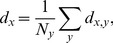
(3)where *d*_x,y_ is the distance of model trace *x* against experimental trace *y*, *N*_y_ is the total number of experimental traces, and *d*_x_ is the distance of model trace *x* against all of the *y* experimental data traces. The total distance *d*_x_ was normalized with the number of experimental traces *N*_y_ so that distances between databases with different *N*_y_ could be meaningfully compared to one another. The consolidated database was then sorted from low to high *d*_x_ values, resulting in a ranking of models from better to poorer representations of O-LM cells, respectively.

### Model database analysis

#### Database cutoff criteria

Two methods of obtaining a subset of O-LM models that best represented experimental data for O-LM cells were developed in this work. The first approach implemented an objective criterion that directly utilized the *d*_x_ distance values. Using the built-in MATLAB function *diff*, the first-order derivative of *d*_x_ was calculated to compare the rate of change of *d*_x_ as a function of the model rank. Nearly identical results were obtained using the multivariate gradient function. Because the distance of a model is a function of one variable – the model's rank in the database – the multivariate gradient therefore reduces to the univariate differences of the distances as a function of the rank, as computed by *diff*. The second criterion provided a counterpoint to the general criterion by considering a limited set of representative electrophysiogical measures for the model traces. The firing frequency during the current injection period and the time constant of a single exponential fit to the hyperpolarization-induced sag were selected as the representative characteristics for the +90 pA and −90 pA traces, respectively. These measures were also plotted as a function of the model rank in the database, and the values were compared to histograms of the measure in the experimental dataset. The cutoff points for each representative measure were obtained as described in Results.

#### Ordering of conductances using high-dimensional visualization algorithm

In order to reduce the total number of pairwise comparisons to be made across all nine voltage-gated ion channel conductances used in this work, a technique for determining, at a glance, how the model outputs changed as a function of the changes in their parameters was applied. This was done using dimensional stacking, a technique for visualizing a high-dimensional space of models in two-dimensions [31]–[33]. See Figure S1 for a dimensional stack image of the subset of ranked models extracted using the general cutoff criterion. The ordering of the parameters in a given “stack” is important in determining sensitivity of model outputs to parameter changes. An optimal stack can be found where the models are organized in the image such that highly-ranked models cluster together more tightly than poorly-ranked models. In this case, the high-order parameters (conductance densities and distributions) in the stack reflect those conductances whose changes in value are associated with changes in model ranking, or goodness-of-fit to experimental data. On the other hand, low-order parameters are those for which changes can be made and yet the ranking of the models are not appreciably affected. Therefore, high-order parameters, or conductance densities and distributions, are those to which the model distances are most sensitive and therefore are the likeliest to demonstrate compensatory balances with each other. To determine this, the high-order parameters were used to construct the conductance histogram images. However, although the first-order parameters in the dimensional stack images were readily considered the highest-order and the fifth-order parameters were readily considered the lowest-order, it was not entirely clear whether the third- and fourth-order parameters should be considered high-order or low-order. Therefore, for the conductance histogram images, third- and fourth-order parameters were also used in order to check whether they showed correlations with other conductances. See Table S3 for the list of high-order conductances as determined by the per-morphology ranked database subsets. Most high-order conductances, especially of the first- and second-orders, were largely shared between the four model database subsets.

#### Verifying passive properties fitting

After the models were ranked against the experimental data as described above, it was important to verify that the passive properties of the model would not appreciably change if refitted using the active conductances exhibited by highly-ranked models. This is because active conductances play a role in the current/voltage dynamics of the experimental protocol used in fitting the passive properties. It was thus conceivable that a model with different maximum conductance densities may have result in a different fit of the passive properties if the maximum conductance densities of its ion channel models were allowed to vary. To ascertain this, two highly-ranked models of each model morphology were taken from the database subset of appropriate O-LM models. In particular, the most highly-ranked model of each morphology with somatodendritic h-currents were found and used to re-fit the passive properties. This is because h-current in both somatic and dendritic compartments cover a much greater surface of the cell's membrane and can therefore more strongly affect the model's membrane response to the passive properties experimental protocol. The two models obtained were rank 1 from the morphology 1-specific subset of the aggregate distance ranking and rank 3 from the morphology 2-specific subset of the aggregate distance ranking. The parameters for these two models are shown in Table S4. The same protocol described for the fitting of passive properties to experimental data, above, was used in re-fitting the passive properties using these two models. After the fitting procedure was completed, the passive membrane properties of the two models were compared to those obtained from the reference model. Table S5 shows the re-fit passive properties of the highly-ranked morphology 1 and morphology 2 models. Note that the passive properties did not seem to vary appreciably as compared to the originally fit values in Table 1. To verify that the differences in passive properties did not significantly affect the model behaviour, the voltage traces of the two highly-ranked models before and after fitting of the passive properties were compared (Figure S2; compare with Table 1). The voltage responses were found to be very similar regardless of whether the original or re-fit passive properties were used. Therefore, it was determined that the passive properties obtained by fitting the reference model against the experimental data were adequate for the ensemble of models subsequently obtained, and that it was not necessary to re-evaluate the simulations using the newly fit passive properties.

## Supporting Information

Figure S1
**Dimensional stack image of the highly-ranked O-LM models in the general criterion database subset.** Each coloured point in the image corresponds to a model in the subset; black regions correspond to models that are not included in the subset. See Results of main text for description of general database subset. The ranking of models is reflected in the colour, from highest-ranked (red end of spectrum) to lowest-ranked (blue end of spectrum) of the subset of highly-ranked models. The axes show the ordering of model parameters as obtained by the clutter-based dimension reordering (CBDR) algorithm [31]. The parameters include the maximum conductance densities of all voltage-gated ion channels in the model: 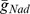, 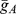, 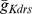, 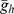, 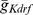, 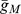, 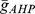, 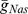, 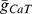, 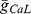, as well as the “cell” parameter which refers to the morphology of the model (one of two possibilities) and the distribution of *I*_h_, also one of two possibilities (0 =  soma only, 1 =  soma and dendrites). The vertical and horizontal lines in the axes show the region of models in the image for which the maximum conductance density labelled in that particular axis is uniform in value. Thus, lower order conductances (small lines, e.g., CaT and CaL) are those for which the maximum conductance density values can change without affecting the ranking of the models, as reflected in the regions of similarly-coloured models that nevertheless possesss different values of those conductances. In all cases, the maximum conductance density values for each axis increase away from the origin in the bottom left-hand corner.(DOC)

Figure S2 **Voltage traces of highly-ranked models corresponding to original and re-fit passive properties.** The blue traces show the model response to +90 pA current injection and with original passive properties, whereas the red traces show the model response to +90 pA current injection and with the re-fit passive properties, for the models with morphology 1, rank 1 (left) and morphology 2, rank 3 (right). As can be seen, the voltage responses are very similar regardless of whether the original or re-fit passive properties were used.
(DOC)

Table S1 **Electrophysiological measurements used in the hyperpolarizing current clamp experimental dataset.** The average values across all experimental voltage traces from application of –90 pA hyperpolarizing current step, as well as the standard deviation of the measures within the dataset, are provided. There were 11 measures used in total.
(DOC)

Table S2 **Electrophysiological measurements used in the depolarizing current clamp experimental dataset.** The average values across all experimental voltage traces from application of +90 pA depolarizing current step, as well as the standard deviation of the measures within the dataset, are provided. There were 92 measures used in total. The nomenclature of the measures follow the pattern of a prefix of one of “*Ini*” (sometimes named “*Spont*”), “*Pulse*”, or “*Recov*” respectively corresponding to whether the measure was calculated for the initial period of the trace prior to the 1s-long current injection step (“*Ini*” or “*Spont*”), or during the current injection step period itself (“*Recov*”), or during the remainder of the trace after the current injection period (“*Recov*”). The rest of the name describes the measure itself, and the suffixes of “*Mean*” and “*Mode*” denote the means and modes, respectively, of all the times the measure was sampled for the given period. For instance, *PulseSpikeMinVmMean* denotes the mean of the minimum achieved somatic *V*_m_ for all spikes in the current injection period. Some measures do not have associated statistical measures, such as *PulseSpikes*, which is simply the number of spikes during the current injection period.
(DOC)

Table S3 **High-order parameters as determined by dimensional stacking analysis.** The ranked database is subdivided into eight subsets according to lines of morphology, *I*_h_ distribution, and cutoff criterion. The ordering of conductances is mostly preserved across all cases. In particular, most high-order conductances, especially of the first- and second-orders, are largely shared between the four model database subsets. This is an indication that the conductances that are important for determining O-LM model output do not critically depend on morphology or distribution of *I*_h_ along soma or dendrites. Furthermore, the high-order conductances do not appreciably change according to the cutoff criterion used for determining the subset of appropriate O-LM models. This is one indication that the general criterion, corresponding to the more inclusive subset of highly-ranked O-LM models, is adequate for delineating a set of appropriate O-LM models that can then be used in analyzing conductance density balances.
(DOC)

Table S4 **Model parameters for the two highest-ranked per-morphology models with somatodendritic h-current.**
(DOC)

Table S5 **Re-fit passive properties for the highly-ranked morphology 1 and morphology 2 models.** Compare with the values fitted prior to the construction of the model database, in Table 2.
(DOC)

Dataset S1 **Experimental current-clamp traces from mouse O-LM cells.** These traces were used in this ensemble modeling work to constrain the models (see Methods). Both +90 pA and -90 pA traces are provided in MATLAB file format.
(ZIP)
