# Supplementary material for: Using Multi-Compartment Ensemble Modeling As an Investigative Tool of Spatially Distributed Biophysical Balances: Application to Hippocampal Oriens-Lacunosum/Moleculare (O-LM) Cells
Source: PLoS One. 2014 Oct 31;9(10):e106567. doi: 10.1371/journal.pone.0106567 (PMC4215854; doi:10.1371/journal.pone.0106567)
Supplement: Figure S1 — Dimensional stack image of the highly-ranked O-LM models in the general criterion database subset. Each coloured point in the image corresponds to a model in the subset; black regions correspond to models that are not included in the subset. See Results of main text for description of general database subset. The ranking of models is reflected in the colour, from highest-ranked (red end of spectrum) to lowest-ranked (blue end of spectrum) of the subset of highly-ranked models. The axes show the ordering of model parameters as obtained by the clutter-based dimension reordering (CBDR) algorithm [31]. The parameters include the maximum conductance densities of all voltage-gated ion channels in the model: , , , , , , , , , , as well as the “cell” parameter which refers to the morphology of the model (one of two possibilities) and the distribution of I h, also one of two possibilities (0 = soma only, 1 = soma and dendrites). The vertical and horizontal lines in the axes show the region of models in the image for which the maximum conductance density labelled in that particular axis is uniform in value. Thus, lower order conductances (small lines, e.g., CaT and CaL) are those for which the maximum conductance density values can change without affecting the ranking of the models, as reflected in the regions of similarly-coloured models that nevertheless possesss different values of those conductances. In all cases, the maximum conductance density values for each axis increase away from the origin in the bottom left-hand corner. (DOC) [file pone.0106567.s001.doc]

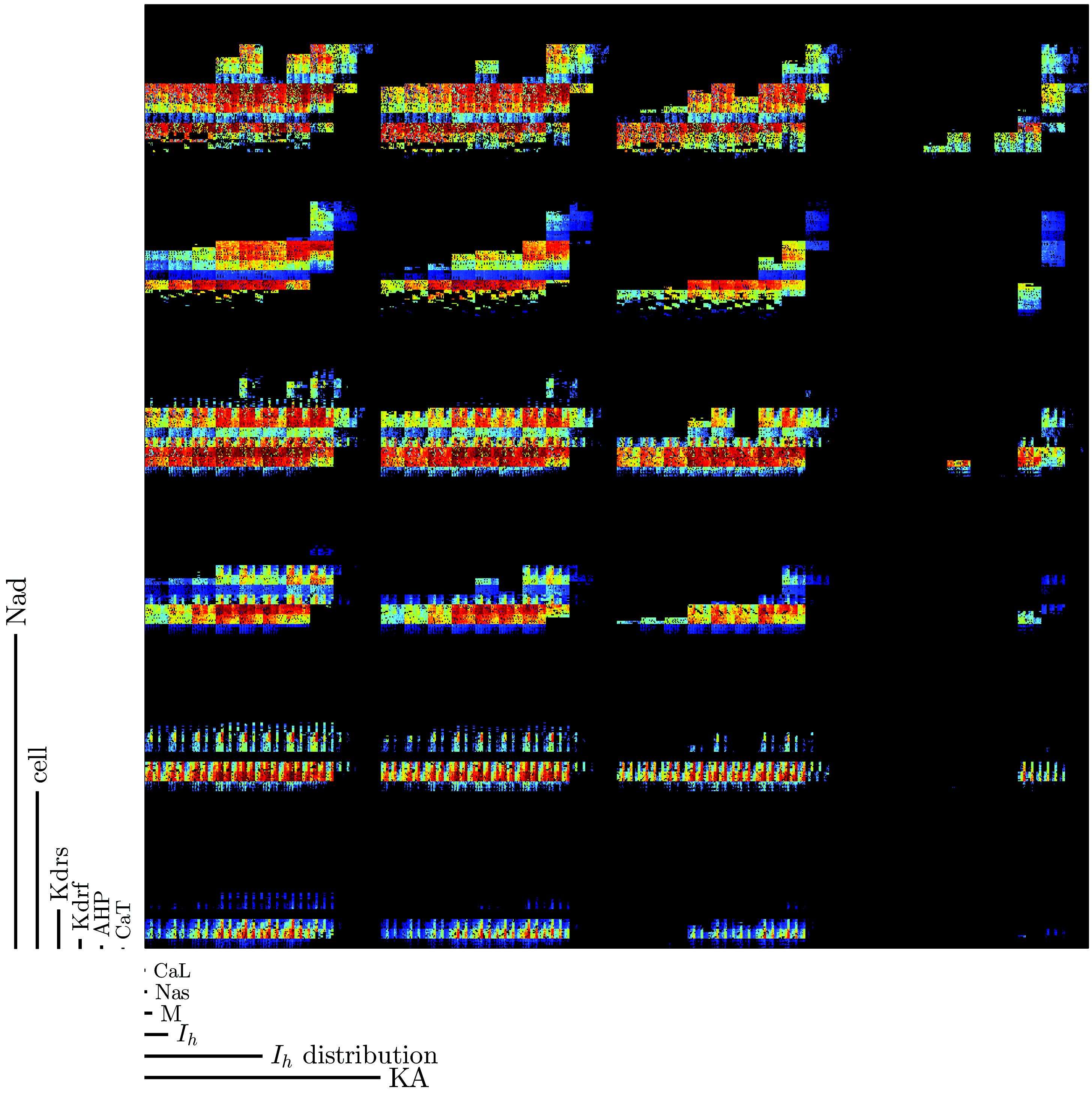


**Figure S1. Dimensional stack image of the highly-ranked O-LM models in the general criterion database subset.** Each coloured point in the image corresponds to a model in the subset; black regions correspond to models that are not included in the subset. See Results of main text for description of general database subset. The ranking of models is reflected in the colour, from highest-ranked (red end of spectrum) to lowest-ranked (blue end of spectrum) of the subset of highly-ranked models. The axes show the ordering of model parameters as obtained by the clutter-based dimension reordering (CBDR) algorithm [31]. The parameters include the maximum conductance densities of all voltage-gated ion channels in the model: $\bar{g}_{Nad}$, $\bar{g}_{A}$, $\bar{g}_{Kdrs}$, $\bar{g}_{h}$, $\bar{g}_{Kdrf}$, $\bar{g}_{M}$, $\bar{g}_{AHP}$, $\bar{g}_{Nas}$, $\bar{g}_{CaT}$, $\bar{g}_{CaL}$, as well as the “cell” parameter which refers to the morphology of the model (one of two possibilities) and the distribution of *I*_h_, also one of two possibilities (0 = soma only, 1 = soma and dendrites). The vertical and horizontal lines in the axes show the region of models in the image for which the maximum conductance density labelled in that particular axis is uniform in value. Thus, lower order conductances (small lines, e.g., CaT and CaL) are those for which the maximum conductance density values can change without affecting the ranking of the models, as reflected in the regions of similarly-coloured models that nevertheless possesss different values of those conductances. In all cases, the maximum conductance density values for each axis increase away from the origin in the bottom left-hand corner.
