# Supplementary material for: Using Multi-Compartment Ensemble Modeling As an Investigative Tool of Spatially Distributed Biophysical Balances: Application to Hippocampal Oriens-Lacunosum/Moleculare (O-LM) Cells
Source: PLoS One. 2014 Oct 31;9(10):e106567. doi: 10.1371/journal.pone.0106567 (PMC4215854; doi:10.1371/journal.pone.0106567)
Supplement: Figure S2 — Voltage traces of highly-ranked models corresponding to original and re-fit passive properties. The blue traces show the model response to +90 pA current injection and with original passive properties, whereas the red traces show the model response to +90 pA current injection and with the re-fit passive properties, for the models with morphology 1, rank 1 (left) and morphology 2, rank 3 (right). As can be seen, the voltage responses are very similar regardless of whether the original or re-fit passive properties were used. (DOC) [file pone.0106567.s002.doc]

**Figure S2. Voltage traces of highly-ranked models corresponding to original and re-fit passive properties.** The blue traces show the model response to +90 pA current injection and with original passive properties, whereas the red traces show the model response to +90 pA current injection and with the re-fit passive properties, for the models with morphology 1, rank 1 (left) and morphology 2, rank 3 (right). As can be seen, the voltage responses are very similar regardless of whether the original or re-fit passive properties were used.
