# Supplementary material for: Using Multi-Compartment Ensemble Modeling As an Investigative Tool of Spatially Distributed Biophysical Balances: Application to Hippocampal Oriens-Lacunosum/Moleculare (O-LM) Cells
Source: PLoS One. 2014 Oct 31;9(10):e106567. doi: 10.1371/journal.pone.0106567 (PMC4215854; doi:10.1371/journal.pone.0106567)
Supplement: Table S1 — Electrophysiological measurements used in the hyperpolarizing current clamp experimental dataset. The average values across all experimental voltage traces from application of –90 pA hyperpolarizing current step, as well as the standard deviation of the measures within the dataset, are provided. There were 11 measures used in total. (DOC) [file pone.0106567.s003.doc]

| **Measure** | **Meaning** | **Average ± Standard deviation** |
| --- | --- | --- |
| *IniSpontPotAvg* | The average *V*_m_ value for the duration of the spontaneous (pre-current injection) period. (mV) | -74.1 ± 0.6 |
| *IniSpontPotRange* | The range of *V*_m_ values for the duration of the spontaneous period. It is important to differentiate between models that have the same average *V*_m_ but large variance, since most experimental traces have little variance in their initial *V*_m_ values prior to the current injection period. (mV) | 0.5 ± 0.1 |
| *RecSpontPotAvg* | The average *V*_m_ value for the duration of the recovery (post-current injection) period. (mV) | -70.4 ± 1.4 |
| *PulsePotMin* | The minimum value of *V*_m_ obtained during the current injection period. I.e., the minimum membrane voltage induced by the hyperpolarization. (mV) | -113.9 ± 3.7 |
| *PulsePotMinTime* | The time at which the minimum of *V*_m_ occurred during the current injection period. (ms) | 166.8 ± 54.7 |
| *PulsePotSag* | The amount of sag (in mV) exhibited by the trace as a result of the hyperpolarization. This is a measure of the depolarizing effects of the *I*_h_ current. (mV) | 14.2 ± 3.1 |
| *PulsePotTau* | The time constant for fitting an exponential curve to the decay of the hyperpolarization-induced sag. (ms) | 47.4 ± 8.0 |
| *RecSpont1SpikeRate* | The firing frequency of the model or experimental cell in the first half of the recovery period. (Hz) | 1.9 ± 2.0 |
| *RecSpont1SpikeRateISI* | The inter-spike interval of the spikes in the first half of the recovery period. (ms) | 2.4 ± 3.2 |
| *RecovSpikes* | The number of spikes in the recovery period. It was not appropriate to use the *RecovSpikeRate*, or firing frequency for the entire duration of the recovery period, because most model and experimental cells exhibited a limited number of post-inhibitory rebound spikes, which were primarily confined to the initial portion of the recovery period. (Number of spikes) | 0.9 ± 1.0 |
| *Ihold* | The amount of holding current that was applied to keep the model or experimental cell’s membrane potential at −74 mV. (nA) | -1.6 ± 6.7 |

**Table S1. Electrophysiological measurements used in the –90pA current clamp experimental dataset.** The average values across all experimental voltage traces from application of
–90pA hyperpolarizing current step, as well as the standard deviation of the measures within the dataset, are provided. There were 11 measures used in total.
