# Supplementary material for: Using Multi-Compartment Ensemble Modeling As an Investigative Tool of Spatially Distributed Biophysical Balances: Application to Hippocampal Oriens-Lacunosum/Moleculare (O-LM) Cells
Source: PLoS One. 2014 Oct 31;9(10):e106567. doi: 10.1371/journal.pone.0106567 (PMC4215854; doi:10.1371/journal.pone.0106567)
Supplement: Table S2 — Electrophysiological measurements used in the depolarizing current clamp experimental dataset. The average values across all experimental voltage traces from application of +90 pA depolarizing current step, as well as the standard deviation of the measures within the dataset, are provided. There were 92 measures used in total. The nomenclature of the measures follow the pattern of a prefix of one of “Ini” (sometimes named “Spont”), “Pulse”, or “Recov” respectively corresponding to whether the measure was calculated for the initial period of the trace prior to the 1s-long current injection step (“Ini” or “Spont”), or during the current injection step period itself (“Recov”), or during the remainder of the trace after the current injection period (“Recov”). The rest of the name describes the measure itself, and the suffixes of “Mean” and “Mode” denote the means and modes, respectively, of all the times the measure was sampled for the given period. For instance, PulseSpikeMinVmMean denotes the mean of the minimum achieved somatic V m for all spikes in the current injection period. Some measures do not have associated statistical measures, such as PulseSpikes, which is simply the number of spikes during the current injection period. (DOC) [file pone.0106567.s004.doc]

| **Measure** | **Meaning** | **Average ± Standard deviation** |
| --- | --- | --- |
| *IniSpontPotAvg* | Average *V*_m_ during the initial or recovery periods of the trace. (mV) | -74.6 ± 0.9 |
| *IniSpontPotRange* | Difference between the maximum and minimum *V*_m_ values during the initial period. (mV) | 0.6 ± 0.2 |
| *IniSpontSpikeRate* | Firing rate of the given period of the trace. (Hz) | 0 ± 0 |
| *IniSpontSpikeRateISI* | Averaged inter-spike-interval (ISI) across the entire period of the trace. (ms) | 0 ± 0 |
| *PulseFirstISI* | First ISI in the pulse period; i.e., the time between the first and second spikes in the pulse period. (ms) | 33.3 ± 8.6 |
| *PulseFirstSpikeTime* | Amount of time between the start of the pulse period and the first spike (delay to first spike; ms). | 19.5 ± 5.7 |
| *PulseISICV* | Coefficient of variation of inter-spike-intervals (ISI) within the pulse period. Normalized (STD/mean) | 0.07 ± 0.04 |
| *PulseIni100msISICV* | Coefficient of variation of inter-spike-intervals (ISI) within the first 100ms of the pulse period. Normalized (STD/mean) | 0.02 ± 0.01 |
| *PulseIni100msRest1SpikeRate* | Firing rate of the first half of the remainder of the pulse period following the initial 100ms. (Hz) | 29.6 ± 6.4 |
| *PulseIni100msRest1SpikeRateISI* | Averaged inter-spike-interval (ISI) of the first half of the remainder of the pulse period following the initial 100ms. (ms) | 29.9 ± 6.7 |
| *PulseIni100msRest2SpikeRate* | Firing rate of the second half of the remainder of the pulse period following the initial 100ms. (Hz) | 13.0 ± 7.1 |
| *PulseIni100msRest2SpikeRateISI* | Averaged inter-spike-interval (ISI) of the second half of the remainder of the pulse period following the initial 100ms. (ms) | 26.1 ± 7.2 |
| *PulseIni100msSpikeRate* | Firing rate of the initial 100ms of the pulse period. (Hz) | 30.3 ± 8.6 |
| *PulseIni100msSpikeRateISI* | Averaged inter-spike-interval (ISI) of the initial 100ms of the pulse period. (ms) | 32.1 ± 8.1 |
| *PulsePotAvg* | Average *V*_m_ for the entire pulse period. (mV) | -63.1 ± 3.6 |
| *PulseSFA* | Spike frequency accommodation (SFA) of the inter-spike-intervals (ISI) during the pulse period. (Ratio of ISIs at end of current injection step period to ISIs at beginning.) | 1.2 ± 0.1 |
| *PulseSpikeRate* | Firing frequency during the pulse period. (Hz) | 22.2 ± 3.0 |
| *PulseSpikeRateISI* | Mean inter-spike-interval (ISI) between spikes during the pulse period. (ms) | 29.5 ± 7.0 |
| *RecIniSpontPotRatio* | Ratio of the averaged *V*_m_ of the recovery period to the averaged *V*_m_ of the initial period. (Normalized) | 1.1 ± 0.02 |
| *RecIniSpontRateRatio* | Ratio of the firing rage of the recovery period to the firing rate of the initial period. (Normalized from Hz) | 1.1 ± 0.32 |
| *RecSpont1SpikeRate* | Mean firing rate of the first half of the recovery period. (Hz) | 0.05 ± 0.32 |
| *RecSpont1SpikeRateISI* | Averaged inter-spike-interval (ISI) of the first half of the recovery period. (ms) | 0.1 ± 0.3 |
| *RecSpont2SpikeRate* | Mean firing rate of the second half of the recovery period. (Hz) | 0 ± 0 |
| *RecSpont2SpikeRateISI* | Averaged inter-spike-interval (ISI) of the second half of the recovery period. (ms) | 0 ± 0 |
| *RecSpontPotAvg* | mV | -78.3 ± 1.4 |
| *RecSpontSpikeRate* | Hz | 0.03 ± 0.2 |
| *RecSpontSpikeRateISI* | ms | 0.02 ± 0.16 |
| *IniSpikes* | Number of spikes in the given period. | 0 ± 0 |
| *SpontSpikeAmplitudeMode* | The mode of the amplitude of the spikes during the current injection period. Amplitude is calculated by taking the difference in spike height from the *V*_m_ at spike initiation. Spike initiation is determined by finding the point of maximum curvature in the V-dV/dt phase plane (PANDORA supports additional methods for spike initiation detection). (mV) | 0 ± 0 |
| *SpontSpikeBaseWidthMode* | Width of the base of spikes, averaged across all spikes in a period. (ms) | 0 ± 0 |
| *SpontSpikeDAHPMagMode* | Magnitude of the double afterhyperpolarization (AHP) peak, if any, where a double AHP indicates the presence of a second afterhyperpolarization (AHP) peak after an initial AHP following the termination of a spike. (mV) | 0 ± 0 |
| *SpontSpikeFallTimeMode* | Time a spike takes to fall from its peak back to the spike initiation point, averaged across all spikes in a period. (ms) | 0 ± 0 |
| *SpontSpikeFixVWidthMode* | Width of spikes at a particular *V*_m_ value (default of –10 mV), averaged across all spikes in a period. (ms) | 0 ± 0 |
| *SpontSpikeHalfVmMode* | *V*_m_ equal to half of the spike height, averaged across all spikes in a period. (mV) | 0 ± 0 |
| *SpontSpikeHalfWidthMode* | Width of spike at the point where *V*_m_ is equal to half of the spike height, averaged across all spikes in a period. (ms) | 0 ± 0 |
| *SpontSpikeInitTimeMode* | Time at initiation of a spike. (ms) | 0 ± 0 |
| *SpontSpikeInitVmBySlopeMode* | *V*_m_ at point of spike initialization, taken to be at the point where the first-order derivative of *V*_m_ exceeds a threshold (default dV/dt threshold of 15 mV/s). (mV) | 0 ± 0 |
| *SpontSpikeInitVmMode* | Default measure finding the initialization of a spike as the point of maximum curvature in the V^dV^/_dt_ phase plane. (mV) | 0 ± 0 |
| *SpontSpikeMaxAHPMode* | The magnitude of *V*_m_ change from the spike initiation point to the minimum *V*_m_, calculated during the abolishing of a spike. (mV) | 0 ± 0 |
| *SpontSpikeMaxVmSlopeMode* | Maximum slope, or first-order derivative of *V*_m_. (mV/ms) | 0 ± 0 |
| *SpontSpikeMinTimeMode* | Time at which the minimum value of *V*_m_ occurs during a spike, relative to the start of the spike. (ms) | 0 ± 0 |
| *SpontSpikeMinVmMode* | Minimum *V*_m_ during a spike. (mV) | 0 ± 0 |
| *SpontSpikePeakVmMode* | *V*_m_ at the spike height; i.e., maximum *V*_m_. (mV) | 0 ± 0 |
| *SpontSpikeRiseTimeMode* | Time from spike initiation to the maximum *V*_m_, or spike height. (ms) | 0 ± 0 |
| *PulseSpikeAmplitudeMean* | The mean amplitude of the spikes during the current injection period. Amplitude is calculated by taking the difference in spike height from the *V*_m_ at spike initiation. Spike initiation is determined by finding the point of maximum curvature in the V-dV/dt phase plane (PANDORA supports additional methods for spike initiation detection). (mV) | 62.2 ± 6.8 |
| *PulseSpikeAmplitudeMode* | Mode of above measure. (mV) | 60.5 ± 6.2 |
| *PulseSpikeBaseWidthMean* | Width of the base of spikes, averaged across all spikes in a period. (ms) | 2.4 ± 0.5 |
| *PulseSpikeBaseWidthMode* | Mode of above measure. (ms) | 2.3 ± 0.6 |
| *PulseSpikeDAHPMagMode* | Magnitude of the double afterhyperpolarization (AHP) peak, if any, where a double AHP indicates the presence of a second afterhyperpolarization (AHP) peak after an initial AHP following the termination of a spike. (mV) | 2.1 ± 4.8 |
| *PulseSpikeFallTimeMean* | Time a spike takes to fall from its peak back to the spike initiation point, averaged across all spikes in a period. (ms) | 1.5 ± 0.5 |
| *PulseSpikeFallTimeMode* | Mode of above measure. (ms) | 1.4 ± 0.4 |
| *PulseSpikeFixVWidthMode* | Width of spikes at a particular *V*_m_ value (default of –10 mV), averaged across all spikes in a period. (ms) | 0.9 ± 0.3 |
| *PulseSpikeHalfVmMean* | *V*_m_ equal to half of the spike height, averaged across all spikes in a period. (mV) | -20.9 ± 2.4 |
| *PulseSpikeHalfVmMode* | Mode of above measure. (mV) | -21.0 ± 2.5 |
| *PulseSpikeHalfWidthMean* | Width of spike at the point where *V*_m_ is equal to half of the spike height, averaged across all spikes in a period. (ms) | 1.2 ± 0.3 |
| *PulseSpikeHalfWidthMode* | Mode of above measure. (ms) | 1.2 ± 0.3 |
| *PulseSpikeInitTimeMean* | Time at initiation of a spike. (ms) | 6.1 ± 0.2 |
| *PulseSpikeInitTimeMode* | Mode of above measure. (ms) | 6.1 ± 0.2 |
| *PulseSpikeInitVmBySlopeMean* | *V*_m_ at point of spike initialization, taken to be at the point where the first-order derivative of *V*_m_ exceeds a threshold (default dV/dt threshold of 15 mV/s). (mV) | -51.0 ± 1.6 |
| *PulseSpikeInitVmBySlopeMode* | Mode of above measure. (mV) | -50.5 ± 1.8 |
| *PulseSpikeInitVmMean* | Default measure finding the initialization of a spike as the point of maximum curvature in the V^dV^/_dt_ phase plane. (mV) | -52.0 ± 1.6 |
| *PulseSpikeInitVmMode* | Mode of above measure. (mV) | -51.7 ± 1.4 |
| *PulseSpikeMaxAHPMean* | The magnitude of *V*_m_ change from the spike initiation point to the minimum *V*_m_, calculated during the abolishing of a spike. (mV) | 18.0 ± 3.4 |
| *PulseSpikeMaxAHPMode* | Mode of above measure. (mV) | 17.3 ± 3.8 |
| *PulseSpikeMaxVmSlopeMean* | Maximum slope, or first-order derivative of *V*_m_. (mV) | 133.4 ± 29.5 |
| *PulseSpikeMaxVmSlopeMode* | Mode of above measure. (mV) | 121.6 ± 28.1 |
| *PulseSpikeMinTimeMean* | Time at which the minimum value of *V*_m_ occurs during a spike, relative to the start of the spike. (ms) | 12.8 ± 1.7 |
| *PulseSpikeMinTimeMode* | Mode of above measure. (ms) | 12.1 ± 1.5 |
| *PulseSpikeMinVmMean* | Minimum *V*_m_ during a spike. (mV) | -69.7 ± 4.1 |
| *PulseSpikeMinVmMode* | Mode of above measure. (mV) | -69.3 ± 5.1 |
| *PulseSpikePeakVmMean* | *V*_m_ at the spike height; i.e., maximum *V*_m_. (mV) | 10.2 ± 5.7 |
| *PulseSpikePeakVmMode* | Mode of above measure. (mV) | 9.3 ± 5.1 |
| *PulseSpikeRiseTimeMean* | Time from spike initiation to the maximum *V*_m_, or spike height. (ms) | 1.0 ± 0.2 |
| *PulseSpikeRiseTimeMode* | Mode of above measure. (ms) | 1.0 ± 0.2 |
| *PulseSpikes* | Number of spikes. | 22.2 ± 3.0 |
| *RecovSpikeAmplitudeMode* | The mode of the amplitude of the spikes during the current injection period. Amplitude is calculated by taking the difference in spike height from the *V*_m_ at spike initiation. Spike initiation is determined by finding the point of maximum curvature in the V-dV/dt phase plane (PANDORA supports additional methods for spike initiation detection). (mV) | 1.6 ± 9.9 |
| *RecovSpikeBaseWidthMode* | Width of the base of spikes, averaged across all spikes in a period. (ms) | 0.1 ± 0.5 |
| *RecovSpikeDAHPMagMode* | Magnitude of the double afterhyperpolarization (AHP) peak, if any, where a double AHP indicates the presence of a second afterhyperpolarization (AHP) peak after an initial AHP following the termination of a spike. (mV) | 0 ± 0 |
| *RecovSpikeFallTimeMode* | Time a spike takes to fall from its peak back to the spike initiation point, averaged across all spikes in a period. (ms) | 0.05 ± 0.32 |
| *RecovSpikeFixVWidthMode* | Width of spikes at a particular *V*_m_ value (default of –10 mV), averaged across all spikes in a period. (ms) | 0.04 ± 0.3 |
| *RecovSpikeHalfVmMode* | *V*_m_ equal to half of the spike height, averaged across all spikes in a period. (mV) | -0.5 ± 2.9 |
| *RecovSpikeHalfWidthMode* | Width of spike at the point where *V*_m_ is equal to half of the spike height, averaged across all spikes in a period. (ms) | 0.04 ± 0.27 |
| *RecovSpikeInitTimeMode* | Time at initiation of a spike. (ms) | 0.02 ± 0.13 |
| *RecovSpikeInitVmBySlopeMode* | *V*_m_ at point of spike initialization, taken to be at the point where the first-order derivative of *V*_m_ exceeds a threshold (default dV/dt threshold of 15 mV/s). (mV) | -1.2 ± 7.8 |
| *RecovSpikeInitVmMode* | Default measure finding the initialization of a spike as the point of maximum curvature in the V^dV^/_dt_ phase plane. (mV) | -1.2 ± 7.8 |
| *RecovSpikeMaxAHPMode* | The magnitude of *V*_m_ change from the spike initiation point to the minimum *V*_m_, calculated during the abolishing of a spike. (mV) | 0.7 ± 4.2 |
| *RecovSpikeMaxVmSlopeMode* | Maximum slope, or first-order derivative of *V*_m_. (mV) | 2.2 ± 14.0 |
| *RecovSpikeMinTimeMode* | Time at which the minimum value of *V*_m_ occurs during a spike, relative to the start of the spike. (ms) | 0.7 ± 4.2 |
| *RecovSpikeMinVmMode* | Minimum *V*_m_ during a spike. (mV) | -1.9 ± 12.0 |
| *RecovSpikePeakVmMode* | *V*_m_ at the spike height; i.e., maximum *V*_m_. (mV) | 0.3 ± 2.1 |
| *RecovSpikeRiseTimeMode* | Time from spike initiation to the maximum *V*_m_, or spike height. (ms) | 0.03 ± 0.2 |
| *RecovSpikes* | Number of spikes. | 0.03 ± 0.16 |

**Table S2. Electrophysiological measurements used in the depolarizing current clamp experimental dataset.** The average values across all experimental voltage traces from application of +90pA depolarizing current step, as well as the standard deviation of the measures within the dataset, are provided. There were 92 measures used in total. The nomenclature of the measures follow the pattern of a prefix of one of “*Ini*” (sometimes named “*Spont*”), “*Pulse*”, or “*Recov*” respectively corresponding to whether the measure was calculated for the initial period of the trace prior to the 1s-long current injection step (“*Ini*” or “*Spont*”), or during the current injection step period itself (“*Recov*”), or during the remainder of the trace after the current injection period (“*Recov*”). The rest of the name describes the measure itself, and the suffixes of “*Mean*” and “*Mode*” denote the means and modes, respectively, of all the times the measure was sampled for the given period. For instance, *PulseSpikeMinVmMean* denotes the mean of the minimum achieved somatic *V*_m_ for all spikes in the current injection period. Some measures do not have associated statistical measures, such as *PulseSpikes*, which is simply the number of spikes during the current injection period.
