# Supplementary material for: Using Multi-Compartment Ensemble Modeling As an Investigative Tool of Spatially Distributed Biophysical Balances: Application to Hippocampal Oriens-Lacunosum/Moleculare (O-LM) Cells
Source: PLoS One. 2014 Oct 31;9(10):e106567. doi: 10.1371/journal.pone.0106567 (PMC4215854; doi:10.1371/journal.pone.0106567)
Supplement: Table S3 — High-order parameters as determined by dimensional stacking analysis. The ranked database is subdivided into eight subsets according to lines of morphology, I h distribution, and cutoff criterion. The ordering of conductances is mostly preserved across all cases. In particular, most high-order conductances, especially of the first- and second-orders, are largely shared between the four model database subsets. This is an indication that the conductances that are important for determining O-LM model output do not critically depend on morphology or distribution of I h along soma or dendrites. Furthermore, the high-order conductances do not appreciably change according to the cutoff criterion used for determining the subset of appropriate O-LM models. This is one indication that the general criterion, corresponding to the more inclusive subset of highly-ranked O-LM models, is adequate for delineating a set of appropriate O-LM models that can then be used in analyzing conductance density balances. (DOC) [file pone.0106567.s005.doc]

|  | **Cell 1, *g*_h_  soma only** | | **Cell 1, *g*_h_ soma and dendrites** | | **Cell 2, *g*_h_  soma only** | | **Cell 2, *g*_h_ soma and dendrites** | |
| --- | --- | --- | --- | --- | --- | --- | --- | --- |
|  | **Orders 1-2** | **Orders 3-4** | **Orders 1-2** | **Orders 3-4** | **Orders 1-2** | **Orders 3-4** | **Orders 1-2** | **Orders 3-4** |
| **Conductances in general model subset** | *g*_Nad_ *g*_KDRf_ *g*_KDRs_ *g*_A_ | *g*_h_ *g*_Nas_ *g*_M_ *g*_AHP_ | *g*_Nad_ *g*_h_ *g*_KDRs_ *g*_A_ | *g*_KDRf_ *g*_Nas_ *g*_M_ *g*_AHP_ | *g*_Nad_ *g*_KDRf_ *g*_KDRs_ *g*_A_ | *g*_h_ *g*_Nas_ *g*_M_ *g*_AHP_ | *g*_Nad_ *g*_h_ *g*_KDRs_ *g*_A_ | *g*_KDRf_ *g*_Nas_ *g*_M_ *g*_AHP_ |
| **Conductances in restricted model subset** | *g*_Nad_ *g*_KDRf_ *g*_KDRs_ *g*_A_ | *g*_h_ *g*_Nas_ *g*_M_ *g*_AHP_ | *g*_Nad_ *g*_h_ *g*_KDRf_ *g*_KDRs_ | *g*_A_ *g*_Nas_ *g*_M_ *g*_AHP_ | *g*_Nad_ *g*_KDRf_ *g*_KDRs_ *g*_A_ | *g*_h_ *g*_Nas_ *g*_M_ *g*_AHP_ | *g*_Nad_ *g*_h_ *g*_KDRf_ *g*_KDRs_ | *g*_A_ *g*_Nas_ *g*_M_ *g*_AHP_ |

Table S3. High-order parameters as determined by dimensional stacking analysis. The ranked database is subdivided into eight subsets according to lines of morphology, *I*_h_ distribution, and cutoff criterion. The ordering of conductances is mostly preserved across all cases. In particular, most high-order conductances, especially of the first- and second-orders, are largely shared between the four model database subsets. This is an indication that the conductances that are important for determining O-LM model output do not critically depend on morphology or distribution of *I*_h_ along soma or dendrites. Furthermore, the high-order conductances do not appreciably change according to the cutoff criterion used for determining the subset of appropriate O-LM models. This is one indication that the general criterion, corresponding to the more inclusive subset of highly-ranked O-LM models, is adequate for delineating a set of appropriate O-LM models that can then be used in analyzing conductance density balances.
