# Supplementary material for: Using Multi-Compartment Ensemble Modeling As an Investigative Tool of Spatially Distributed Biophysical Balances: Application to Hippocampal Oriens-Lacunosum/Moleculare (O-LM) Cells
Source: PLoS One. 2014 Oct 31;9(10):e106567. doi: 10.1371/journal.pone.0106567 (PMC4215854; doi:10.1371/journal.pone.0106567)
Supplement: Table S4 — Model parameters for the two highest-ranked per-morphology models with somatodendritic h-current. (DOC) [file pone.0106567.s006.doc]

| Conductances | ***g*_max_values for model morphology 1, rank 1 (pS/μm^2^)** | *g*_max_ values for model morphology 2, rank 3 (pS/μm^2^) |
| --- | --- | --- |
| *g*_Nad_ | 117 | 230 |
| *g*_Nas_ | 220 | 107 |
| *g*_KDRf_ | 215 | 506 |
| *g*_KDRs_ | 2.3 | 2.3 |
| *g*_KA_ | 2.5 | 32 |
| *g*_h_ (soma + dendrites) | 0.02 | 0.02 |
| *g*_CaL_ | 50 | 25 |
| *g*_CaT_ | 5 | 2.5 |
| *g*_AHP_ | 5.5 | 11 |
| *g*_M_ | 0.375 | 0.75 |

Table S4. Model parameters for the two highest-ranked per-morphology models with somatodendritic h-current. Compare values with the model parameters listed in Table 2.
