# Supplementary material for: Using Multi-Compartment Ensemble Modeling As an Investigative Tool of Spatially Distributed Biophysical Balances: Application to Hippocampal Oriens-Lacunosum/Moleculare (O-LM) Cells
Source: PLoS One. 2014 Oct 31;9(10):e106567. doi: 10.1371/journal.pone.0106567 (PMC4215854; doi:10.1371/journal.pone.0106567)
Supplement: Table S5 — Re-fit passive properties for the highly-ranked morphology 1 and morphology 2 models. Compare with the values fitted prior to the construction of the model database, in Table 2. (DOC) [file pone.0106567.s007.doc]

| **Passive properties** | **Re-fit values for model morphology 1, rank 1** | **Re-fit values for model morphology 2, rank 3** |
| --- | --- | --- |
| *R*_a_ (Ω ⋅ cm) | 300 | 300 |
| *C*_m_ (μF/cm^2^) | 0.96857 | 0.9 |
| *R*_m_ (Ω ⋅ cm^2^) | 61117 | 40397 |
| *E*_L_ (mV) | −71.4 | −68.7 |
| *g*_KL_ (S/cm^2^) | 9.9137^-10^ | 9.9256^-10^ |

Table S5. Re-fit passive properties for the highly-ranked morphology 1 and morphology 2 models. Compare with the values fitted prior to the construction of the model database, in Table 2.
